# A single hole spin with enhanced coherence in natural silicon

**DOI:** 10.1038/s41565-022-01196-z

**Published:** 2022-09-22

**Authors:** N. Piot, B. Brun, V. Schmitt, S. Zihlmann, V. P. Michal, A. Apra, J. C. Abadillo-Uriel, X. Jehl, B. Bertrand, H. Niebojewski, L. Hutin, M. Vinet, M. Urdampilleta, T. Meunier, Y.-M. Niquet, R. Maurand, S. De Franceschi

**Affiliations:** 1 Université Grenoble Alpes, CEA, Grenoble INP, IRIG-Pheliqs, Grenoble, France; 2 Université Grenoble Alpes, CEA, IRIG-MEM-L_Sim, Grenoble, France; 3 Université Grenoble Alpes, CEA, LETI, Minatec Campus, Grenoble, France; 4 Université Grenoble Alpes, CNRS, Grenoble INP, Institut Néel, Grenoble, France

**Keywords:** Qubits, Quantum dots

## Abstract

Semiconductor spin qubits based on spin–orbit states are responsive to electric field excitations, allowing for practical, fast and potentially scalable qubit control. Spin electric susceptibility, however, renders these qubits generally vulnerable to electrical noise, which limits their coherence time. Here we report on a spin–orbit qubit consisting of a single hole electrostatically confined in a natural silicon metal-oxide-semiconductor device. By varying the magnetic field orientation, we reveal the existence of operation sweet spots where the impact of charge noise is minimized while preserving an efficient electric-dipole spin control. We correspondingly observe an extension of the Hahn-echo coherence time up to 88 μs, exceeding by an order of magnitude existing values reported for hole spin qubits, and approaching the state-of-the-art for electron spin qubits with synthetic spin–orbit coupling in isotopically purified silicon. Our finding enhances the prospects of silicon-based hole spin qubits for scalable quantum information processing.

## Abstract (web summary)

Operation sweet spots decouple hole spin qubits in silicon from charge noise while conserving full electrical control and allowing for spin coherence times of up to 88 μs.

## Main

In the global effort to build scalable quantum processors, spin qubits in semiconductor quantum dots^1^ are progressively making their mark^2^. We highlight, in particular, the achievement of single-^3,4^ and two-qubit^5–8^ gate fidelities well above 99%, the first realizations of multi-qubit arrays^9,10^ and a demonstrated compatibility with industrial-grade semiconductor manufacturing technologies^11–13^.

Due to their long coherence time, electron-spin qubits in silicon quantum dots have so far attracted the most attention^2^. That said, their control requires add-ons such as metal microstrips^3^, micromagnets^4^ or dielectric resonators^14^, the large-scale integration of which is technically challenging^13^. Hole spin qubits, on the other hand, can circumvent this difficulty due to their intrinsically large spin–orbit coupling, which enables electric-dipole spin manipulation. Over the last five years a variety of hole spin qubits have been reported in both silicon^11,15^ and germanium^16–19^ quantum dots. In all these qubits, quantum operations are performed using high-frequency gate voltage excitations.

The downside of all-electrical spin control is that the required spin–orbit coupling exposes the qubit to charge noise, leading to a reduced hole spin coherence. Recent theoretical works^20–22^, however, have shown that, for properly chosen structural geometries and magnetic field orientations, careful tuning of the electrostatic confinement can bring the hole qubit to an optimal operation point where the effects of charge noise vanish to first order while enabling efficient electric-dipole spin resonance. Here, using a single hole spin confined in natural silicon, we pinpoint the existence of operation sweet spots where the longitudinal spin-electric susceptibility is minimized, resulting in a large enhancement of the spin coherence time.

Numerical simulations are found in remarkable agreement with the experimental observations, and predict that such sweet spots are resilient to realistic amounts of disorder. This advocates the use of such sweet spots as a reliable way to decouple hole spin qubits from charge noise, thereby reinforcing the promises of emergent hole-based quantum processors^23^.

## Device design and *g*-factor anisotropy

**Fig. 1:**
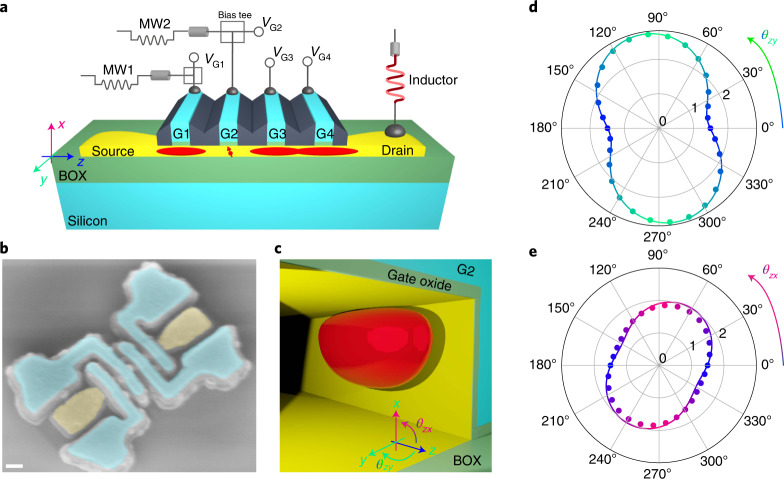
Device, measurement scheme and properties of the first confined hole. **a**, Simplified three-dimensional representation of a silicon (yellow)-on-insulator (green) nanowire device with four gates (light blue) labelled G1, G2, G3 and G4. Gate G2 defines a quantum dot (QD2) hosting a single hole; G3 and G4 define a hole island used as reservoir and sensor for hole spin readout; G1 defines a hole island screening QD2 from dopant disorder and fluctuations in the source. Using bias tees, both static voltages (*V*_G1_, *V*_G2_) and time-dependent, high-frequency voltages (MW1, MW2) can be applied to G1 and G2, respectively. The drain contact is connected to an off-chip, surface-mount inductor to enable radiofrequency reflectometry readout. The coordinate system used for the magnetic field is shown on the left side (in the crystal frame, *x* = [001], $y=[1\bar{1}0]$ and *z* = [110]). Each axis is given a different colour, which is used throughout the manuscript to indicate the magnetic field orientation. **b**, Colourized scanning electron micrograph showing a tilted view of a device similar to the measured one. Image taken just after the etching of the spacer layers. Scale bar, 100 nm. **c**, Rendering of the calculated wave function of the first hole accumulated under G2. **d**, Measured (dots) and calculated (solid line) hole *g*-factor as a function of the in-plane magnetic field angle *θ*_*zy*_ (dots). *θ*_*zy*_ = 90^∘^ corresponds to a magnetic field applied along the *y* axis. **e**, Same as **d** but in the *xz* plane. *θ*_*zx*_ = 90^∘^ corresponds to a magnetic field applied along the *x* axis. BOX, buried oxide.

**Extended Data Fig. 1:**
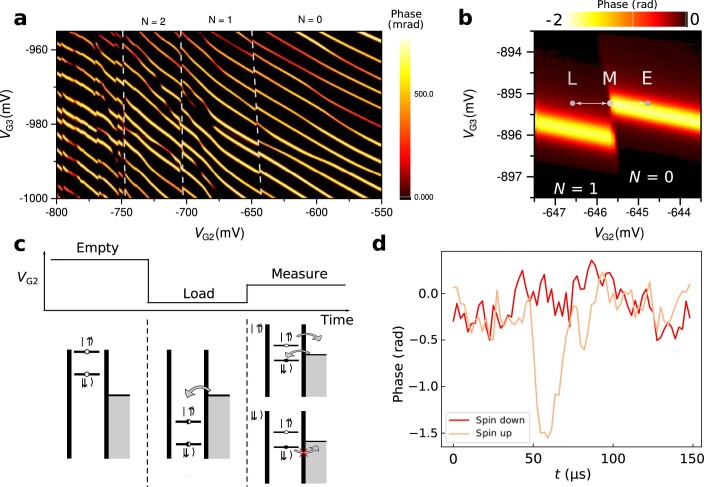
Single shot spin readout. **(a)** Stability diagram of the device as a function of *V*_G2_ and *V*_G3_. The dashed grey lines are guides to the eye highlighting charge transitions in QD2. The first hole tunnels into QD2 at *V*_G2_ ≈ − 650 mV. **(b)** Zoom on the stability diagram close to the working point used in the main text. The points labelled L (Load), M (Measure) and E (Empty) are the three stages of the pulse sequence applied to *V*_G2_ for spin readout. **(c)** (Top) Schematic of the three stages pulse sequence applied to *V*_G2_. (Bottom) Schematic energy diagrams at the different stages of the pulse sequence. *μ*_*F*_ is the chemical potential of the charge sensor playing the role of reservoir. A random spin is charged during the load stage. At the measure stage, if the loaded spin is up, the hole is able to tunnel out and is replaced by a spin down. On the opposite, if the loaded spin is down, tunneling in or out is impossible. Finally, the dot is discharged during the empty stage. **(d)** Phase versus time during the measurement stage. The orange curve exhibits a ‘blip’ around *t* = 50 *μ*s, which indicates that the dot experienced a discharge/charge cycle characteristic of a spin up loading (see c). On the contrary, the red curve shows no phase change, which can be interpreted as a spin down loading. The phase signal is integrated over 6 *μ*s.

Our device consists of an undoped silicon nanowire of rectangular cross section in which the electrostatics is controlled by four gates (G1–G4) as shown in Fig. 1a,b. We define a large hole island below G3 and G4 to be used simultaneously as a reservoir and as a charge sensor for a single hole trapped in a quantum dot, QD2, under G2. Single-shot readout of this hole spin is performed by means of a spin-to-charge conversion technique based on the real-time detection of spin-selective tunnelling to the reservoir, a widely used method often referred to as ‘Elzerman readout’^24^. Tunnelling events are detected by dispersive radiofrequency reflectometry on the charge sensor (see Methods and Extended Data Fig. 1 for technical details).

**Extended Data Fig. 2:**
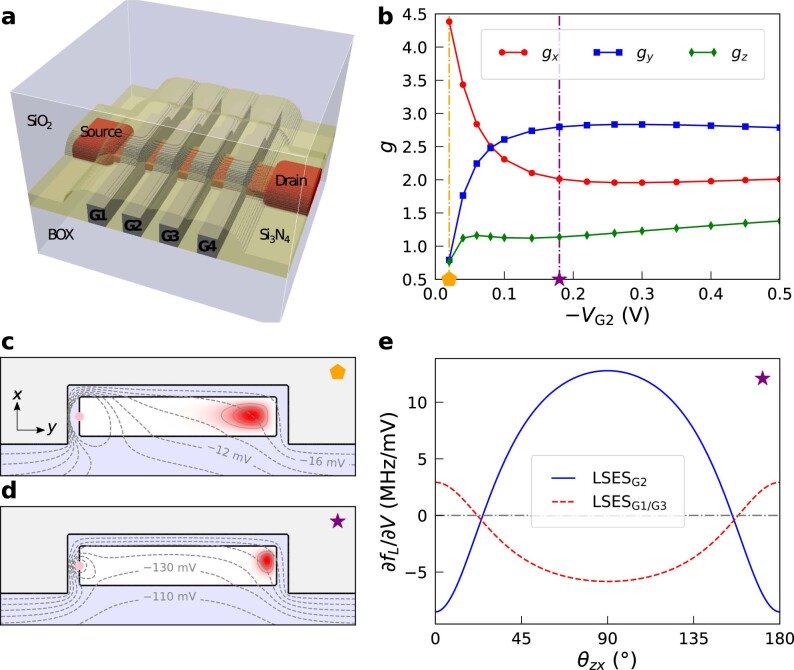
Modeled structure. **(a)** The 17 nm thick and 100 nm wide silicon channel is connected to highly doped source and drain reservoirs and controlled by four gates G1...G4. **(b)** Dependence of the *g*-factors on the electric field in a simple set-up with no hole gases below G1, G3 and G4. The *g*-factors *g*_*x*_, *g*_*y*_, and *g*_*z*_ are plotted as as a function of the difference of potential − *V*_G2_ between gates G2 and gates G1 and G3 (both grounded). When increasing − *V*_G2_, the lateral electric field from the wrap gate squeezes the hole on the side facet of the channel [see panels (c) and (d)]. This strengthens heavy-hole/light-hole mixing, which results in a decrease of *g*_*x*_, and an increase of *g*_*y*_. **(c, d)** Maps of the squared wave functions (red) in the cross section of the channel below gate G2, at the biases marked with an orange pentagon and a purple star in (b). The channel is colored in white, the gate G2 in gray and SiO_2_ in blue. The dashed gray lines are isopotential lines of the dot potential *V*_Q*D*_(**r**), spaced by 2 mV in (c) and by 10 mV in (d). The isodensity surface of the wave function in (d) that encloses 85% of the hole charge is represented in Fig. 1c of the main text. **(e)** LSES computed at the purple star in (b) as a function of *θ*_*zx*_ (for constant Larmor frequency *f*_*L*_ = 17 GHz). In all these calculations, a single positive charge is introduced on the left facet of the channel [pink dot in (c, d)] to lift the degeneracy between the left and right corner dots. Details about the modeling and dependence of the *g*-factors on bias conditions can be found in Supp. Info S1.

**Extended Data Fig. 3:**
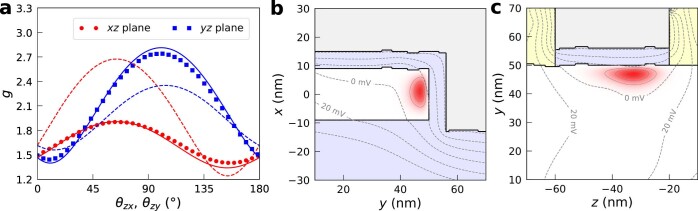
Comparison between the experimental and calculated *g*-factors. **(a)***g*-factors for a magnetic field in the *xz* (red) and *yz* (blue) planes, as a function of the angles *θ*_*zx*_ and *θ*_*zy*_, respectively. The symbols are the experimental data, and the dotted lines are calculated in the pristine device at the experimental bias point (with the hole gases below G1, G3 and G4). The lateral electric field is however too weak at this bias point to match the experimental anisotropy *g*_*y*_ > *g*_*x*_. The solid lines are calculated in a particular realization of a disordered device with roughness and positive charge traps at the Si/SiO_2_ interface and in Si_3_N_4_ (see Methods and Supp. Info S1). These traps tend to strengthen confinement on the side facets (because they are much better screened near the corners of the wrap gate), which increases *g*_*y*_ and decreases *g*_*x*_, as shown in Extended Data Fig. 2. Moreover, *θ*_*zx*_ is shifted by ≈ − 25^∘^ and *θ*_*zy*_ by ≈ 10^∘^ to account for the experimental rotations of the principal axes of the *g*-tensor (resulting from residual strains, see Supp. Info S1). The polar plots of the *g*-factors and the LSES of this disordered device are shown in Figs. 1 and 2 of the main text, respectively. **(b, c)** Maps of the squared wave function (red) computed in the same disordered device, where (b) shows a transverse *xy* cross section at *z* = − 35 nm and (c) a planar *yz* cross-section at *x* = 0. The channel is colored in white, the gate G2 in gray, SiO_2_ in blue and Si_3_N_4_ in yellow. The dashed gray lines are isopotential lines of *V*_Q*D*_(**r**), spaced by 20 mV. *V*_Q*D*_(**r**) is here measured with respect to the energy level of the hole. The robustness of the *g*-factors and operation sweet spots with respect to disorder is discussed in Supp. Info S1.

In our device geometry, the first holes primarily accumulate in the upper corners of the silicon nanowire^25^. Figure 1c displays the expected single-hole wave function in QD2, computed with a finite-differences **k** ⋅ **p** model including the six topmost valence bands^26^ (see Methods and Supplementary Information, section 1). At low energy, that is, close to the valence-band edge, the hole wave function primarily contains heavy-hole (HH) and light-hole (LH) components. The strong two-axes confinement readily seen in Fig. 1c favours HH–LH mixing^27,28^. This mixing is expected to manifest in the anisotropy of the hole *g*-tensor, which carries information on the relative weight of the HH and LH components^29–31^. To verify this, we measure the hole spin resonance frequency *f*_L_ while varying the orientation of the magnetic field **B** in the *xz* and *yz* planes. The effective *g*-factor *g* = *hf*_L_/(*μ*_B_∣**B**∣) (with *μ*_B_ the Bohr magneton and *h* the Planck constant) is plotted in Fig. 1d,e as a function of the magnetic field angles *θ*_*zx*_ and *θ*_*zy*_, respectively. These maps highlight the strong anisotropy of the Zeeman splitting, with a maximal *g* = 2.7 close to the *y* axis (in-plane, perpendicular to the wire) and a minimal *g* = 1.4 close the *z* axis (in-plane, along the wire). The calculated *g*-factors are also plotted in the same figures as coloured solid lines. The agreement with the experimental data is remarkable. From the numerical simulation, we conclude that the measured *g*-factor anisotropy results from a strong electrical confinement against the side facet of the channel (along *y*), which prevails over the mostly structural vertical confinement (along *x*). The experimental *g*-factors and the small misalignment between the principal axes of the *g*-tensor and the device symmetry axes are best reproduced by introducing a moderate amount of charge disorder in combination with small (∼0.1%) shear strains in the silicon channel (Extended Data Figs. 2 and 3, and Supplementary Information, section 1). The latter probably originate from device processing and thermal contraction at the measurement temperature^32^.

## Longitudinal spin-electric susceptibility

Given that the *g*-factor anisotropy is intimately related to the HH–LH mixing, which is controlled by the electrostatic confinement potential, the Larmor frequency is expected to be gate-voltage dependent. As a consequence, the hole spin coherence must be generally susceptible to charge noise. We thus measure the longitudinal spin-electric susceptibility (LSES) with respect to the voltages applied to the lateral gate G1 and to the accumulation gate G2, which we define as ${{{{\rm{LSES}}}}}_{{{{\rm{G1}}}}}=\frac{\partial {f}_{\mathrm{L}}}{\partial {V}_{{{{\rm{G1}}}}}}$ and ${{{{\rm{LSES}}}}}_{{{{\rm{G2}}}}}=\frac{\partial {f}_{\mathrm{L}}}{\partial {V}_{{{{\rm{G2}}}}}}$, respectively. In essence, LSES_G1_ and LSES_G2_ characterize the response of the Larmor frequency to the electric-field components parallel (*z*) and perpendicular (*x*,*y*) to the channel direction, respectively.

**Fig. 2:**
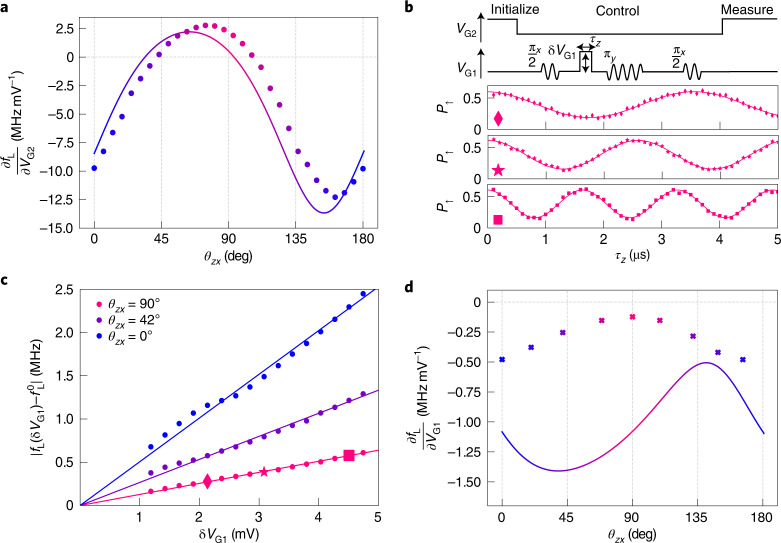
LSES. **a**, Spin-electric susceptibility with respect to *V*_G2_ (LSES_G2_) as a function of magnetic field angle *θ*_*zx*_ (symbols), at constant *f*_L_ = 19 GHz. The LSES vanishes at *θ*_*zx*_ = 41^∘^ and 106^∘^, as indicated by the two arrows. The solid line corresponds to the numerically calculated LSES_G2_. **b**, Top: pulse sequence used to measure LSES_G1_, a voltage pulse of amplitude δ*V*_G1_ and duration *τ*_*z*_ is applied to G1 during the first free evolution time of a Hahn-echo sequence. Bottom: spin-up fraction *P*_↑_ as a function of *τ*_*z*_ for δ*V*_G1_ = 2.16 mV (diamonds), 3.12 mV (stars) and 4.80 mV (squares), at *θ*_*zx*_ = 90^∘^. The oscillation frequency varies with δ*V*_G1_. **c**, δ*V*_G1_ dependence of the frequency shift extracted from the Hahn-echo measurements at *θ*_*zx*_ = 0^∘^, 42^∘^ and 90^∘^. Symbols in the latter data set correspond to the *P*_↑_ oscillations shown in **b**. The solid lines are linear fits to the experimental data whose slope directly yields ∣LSES_G1_∣. **d**, Measured (symbols) and calculated (solid line) LSES_G1_ as a function of *θ*_*zx*_, at constant *f*_L_ = 17 GHz. The negative sign of LSES_G1_ is inferred from the shift of *f*_L_ under a change in *V*_G1_.

**Extended Data Fig. 4:**
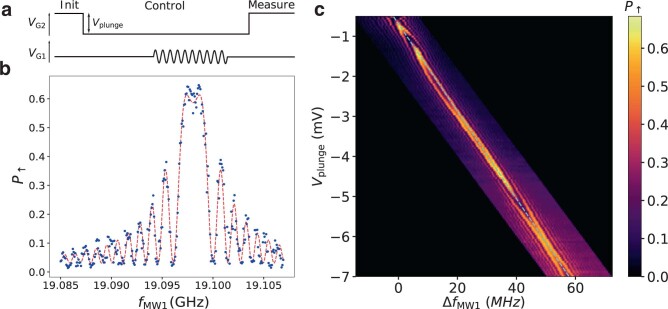
Measurement of LSESG2. **(a)** Schematic representation of the pulse sequence used to monitor spin resonance. We burst on MW1 for 5 *μ*s and average *P*_*↑*_ over 200 such sequences. **(b)** Average *P*_*↑*_ (blue dots) versus MW1 burst frequency at *V*_plunge_ = − 1 mV. This plot is in essence a line cut of a Rabi chevron at *t*_b*urst*_ = 5 *μ*s. The red dashed line is a fit used to extract the Larmor frequency. **(c)** Tracking of *f*_*L*_ as a function of *V*_plunge_. The dashed blue line is a linear fit whose slope is equal to LSES_G2_.

To probe the response to G2, we directly measure the spin resonance frequency *f*_L_ at different *V*_G2_ (Extended Data Fig. 4). The resulting LSES_G2_ is plotted as a function of the magnetic field angle *θ*_*zx*_ in Fig. 2a. The observed angular dependence is in good agreement with the theoretical expectation.

Noticeably, LSES_G2_ is positive along *x* and negative along *z*. Indeed, when increasing *V*_G2_, the hole wave function extends proportionally more in the *yz* plane than in the vertical *x* direction, which increases *g*_*x*_ and decreases *g*_*y*_ and *g*_*z*_ (Extended Data Fig. 2b and Supplementary Information, section 1). As a result of the sign change, LSES_G2_ vanishes at two magnetic field orientations in the *xz* plane (marked by arrows in Fig. 2a), which are sweet spots for electric-field fluctuations perpendicular to the silicon channel.

To probe the response to G1, we introduce a pulse on *V*_G1_ in a Hahn-echo sequence^4^ as outlined in Fig. 2b. This defines a phase gate, controlled by the amplitude δ*V*_G1_ and duration *τ*_*z*_ of the pulse. Figure 2b displays the coherent oscillations recorded as a function of *τ*_*z*_ for three different pulse amplitudes. The frequency of these oscillations is expected to increase linearly with δ*V*_G1_, with a slope ${{{{\rm{LSES}}}}}_{{{{\rm{G1}}}}}=\frac{\partial {f}_{\mathrm{L}}}{\partial {V}_{{{{\rm{G1}}}}}}$. This is shown in Fig. 2c for different magnetic field orientations. LSES_G1_, plotted in Fig. 2d as a function of *θ*_*zx*_, ranges from −0.5 MHz mV^−1^ to −0.1 MHz mV^−1^. Its magnitude is much smaller than that of LSES_G2_ because G1 is further from QD2 than G2 and its field effect is partly screened by the hole gas beneath. The numerically calculated LSES_G1_ (solid line) reproduces reasonably well the order of magnitude but not the angular dependence of the measured LSES_G1_. This discrepancy may be due to inaccuracies in the description of the hole gases near QD2 and to unaccounted charge disorder and strains (see discussion in Supplementary Information, section 1). We also notice that LSES_G1_ never vanishes and that the minimum of ∣LSES_G1_∣ happens to be almost at the same *θ*_*zx*_ as a zero of LSES_G2_.

## Coherence times and frequency-dependent noise contributions

**Fig. 3:**
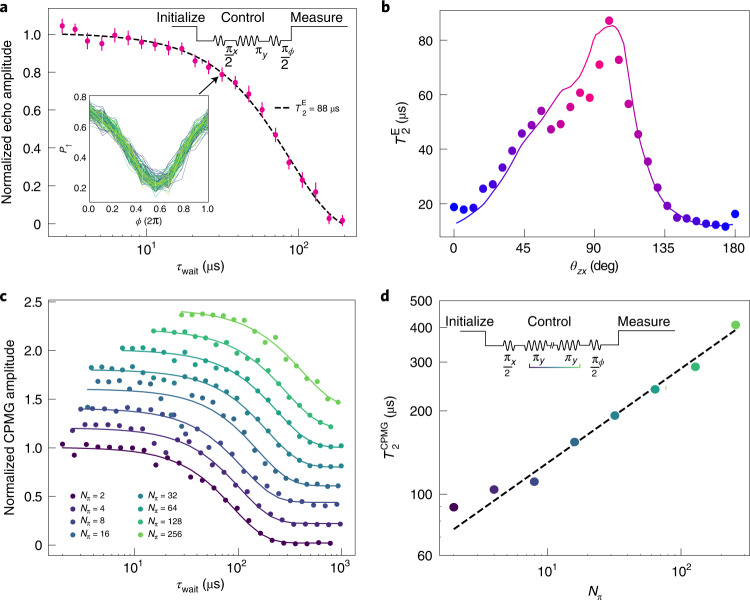
Anisotropy of the hole spin coherence and sweet-spot operation. **a**, Normalized Hahn-echo amplitude versus free evolution time *τ*_wait_ at *f*_L_ = 17 GHz. The top-right inset sketches the pulse sequence. The bottom-left inset displays *P*_↑_ (*τ*_wait_ = 31.4 μs) versus the phase *ϕ* of the last π/2 pulse for 100 repetitions. For each *τ*_wait_, we extract the average amplitude of the *P*_↑_(*ϕ*) oscillations and normalize it to the average amplitude in the zero-delay limit. The resulting normalized echo amplitudes are reported on the main plot. The dashed curve is a fit to $\exp (-{({\tau }_{{{{\rm{wait}}}}}/{T}_{2}^{\mathrm{E}})}^{\beta })$ with *β* = 1.5 ± 0.1. **b**, Measured ${T}_{2}^{{{{\rm{E}}}}}$ versus magnetic field angle *θ*_*zx*_ (symbols). The solid line is a fit to equation (1), using the experimental LSES_G1_ and LSES_G2_ from Fig. 2a–d. **c**, Normalized CPMG amplitude as a function of free evolution time *τ*_wait_ for different numbers *N*_π_ of π pulses (curves are offset for clarity). The solid lines are fits to the same exponential decay function as in **a** with *β* = 1.5. **d**, Extracted ${T}_{2}^{{{{\rm{CPMG}}}}}$ as a function of *N*_π_. The dashed line is a linear fit with slope *γ* = 0.34. The inset sketches the CPMG pulse sequence: *N*_π_ equally spaced π_*y*_ pulses between two π_*x*_/2 pulses. For the Hahn-echo, we detune the phase of the last pulse.

We now turn to the angular dependence of the hole spin coherence time and investigate its correlation with the longitudinal spin-electric susceptibility^33^. To get rid of low-frequency noise sources, we measure the coherence time using a conventional Hahn-echo protocol^2^. The control sequence, applied to G1 (see upper inset of Fig. 3a), consists of π_*x*_/2, π_*y*_ and π_*ϕ*_/2 pulses separated by a time delay *τ*_wait_/2. For each *τ*_wait_, we extract the averaged amplitude of the *P*_↑_ oscillation obtained by varying the phase *ϕ* of the last π/2 pulse, and normalize it to the *P*_↑_ oscillation amplitude in the zero-delay limit.

A representative Hahn-echo plot is shown in Fig. 3a. We fit the echo amplitude to an exponential decay $\exp (-{({\tau }_{{{{\rm{wait}}}}}/{T}_{2}^{{{{\rm{E}}}}})}^{\beta })$, where the exponent *β* is left as a free parameter. The best fit is obtained for *β* = 1.5 ± 0.1, which implies a high-frequency noise with a characteristic spectrum $S(f)={S}_{{{{\rm{hf}}}}}{({f}_{0}/f)}^{\alpha }$, where *f*_0_ = 1 Hz is a reference frequency and *α* = *β* − 1 ≈ 0.5 (we note that the same *α* value was reported for hole spin qubits in germanium^10^).

**Extended Data Fig. 5:**
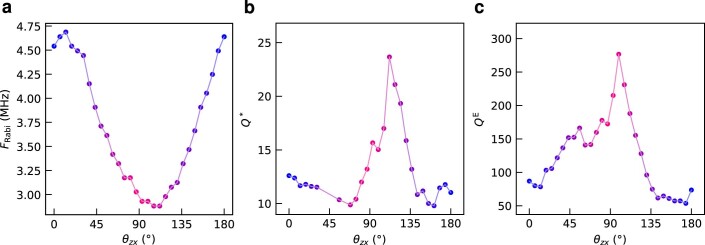
Rabi frequencies and quality factors. **(a)** Rabi frequency as a function of magnetic field orientation *θ*_*zx*_. The Larmor frequency *f*_*L*_ = 17 GHz is kept constant and the hole spin is manipulated by a microwave burst on gate G1 with power *P*_M*W*1_ = 15 dBm on top of the MW1 line. **(b)** Inhomogeneous quality factor ${Q}^{* }={F}_{{{{\rm{R}}}}abi}\times {T}_{2}^{* }$ as a function of the magnetic field orientation *θ*_*zx*_. The data are calculated from the Rabi frequencies plotted in (a), and from the values of ${\overline{T}}_{2}^{* }$ measured for *t*_m*eas*_ = 5.5 s (Fig. 4c). **(c)** Same as (b) for the echo quality factor ${Q}^{{{{\rm{E}}}}}={F}_{{{{\rm{R}}}}abi}\times {T}_{2}^{{{{\rm{E}}}}}$. In the present case, the Rabi frequency is minimal around the sweet spot (a). Nonetheless, the quality factors Q^*^ and *Q*^E^ do peak near the sweet spot owing to the much improved coherence times. They reach Q^*^ = 23 and *Q*^E^ = 276, with peak-to-valley ratios of respectively ≈ 2.5 and ≈ 5.5. As discussed in section Supp. Info S2, we can achieve Rabi frequencies of at least 5 MHz at the sweet spot with a larger driving power *P*_M*W*1_ = 20 dBm, which results in Q^*^ ≈ 35 and *Q*^E^ ≈ 440. In principle, the quality factors may be further improved by driving with gate G2 and looking for the sweet spot in the *xy* plane (see Supp. Info. S1).

To explore the angular dependence of ${T}_{2}^{{{{\rm{E}}}}}$ in the *xz* plane, we measure the decay of the Hahn-echo amplitude for different values of *θ*_*zx*_. The results, shown in Fig. 3b, reveal a strong anisotropy, with ${T}_{2}^{{{{\rm{E}}}}}$ ranging from 15 μs to 88 μs. Strikingly, the spin coherence time peaks at *θ*_*zx*_ = 99°, an angle between the minimum of ∣LSES_G1_∣ and a zero of LSES_G2_, highlighting a correlation with the correspondingly suppressed electrical noise. The extended coherence time is much longer than previously reported for hole spin qubits in both silicon (1.5 μs (ref. ^15^)) and germanium (3.8 μs (ref. ^23^))^34^. In addition, we notice that spin control remains efficient at all angles including *θ*_*zx*_ = 99^∘^, where we could readily achieve Rabi frequencies *F*_Rabi_ as large as 5 MHz limited by the attenuation on the microwave line. The echo quality factor ${Q}^{{{{\rm{E}}}}}={F}_{{{{\rm{Rabi}}}}}\times {T}_{2}^{{{{\rm{E}}}}}$ also peaks at *θ*_*zx*_ = 99°, reaching *Q*^E^ ≈ 440 with further room for improvement (Supplementary Information, section 2 and Extended Data Fig. 5).

The observed angular dependence of ${T}_{2}^{{{{\rm{E}}}}}$ can be understood by assuming that the electrical noise is the sum of uncorrelated voltage fluctuations on the different gates G*i* with respective spectral densities ${S}_{{{{\rm{G}}}}i}(f)={S}_{{{{\rm{G}}}}i}^{{{{\rm{hf}}}}}{({f}_{0}/f)}^{0.5}$. Given the Hahn-echo noise filter function, the decoherence rate can then be expressed as (Supplementary Information, section 3):

1 $$\frac{1}{{T}_{2}^{{{{\rm{E}}}}}}\approx 7.8{f}_{0}^{1/3}{\left(\mathop{\sum}\limits_{i}{\left(\frac{\partial {f}_{\mathrm{L}}}{\partial {V}_{{{{\rm{G}}}}i}}\right)}^{2}{S}_{{{{\rm{G}}}}i}^{{{{\rm{hf}}}}}\right)}^{2/3}.$$

Using the longitudinal spin-electric susceptibilities from Fig. 2a–d and leaving the weights ${S}_{{{{\rm{G}}}}i}^{{{{\rm{hf}}}}}$ as adjustable parameters, we achieve a remarkable agreement with the experimental ${T}_{2}^{{{{\rm{E}}}}}$ (coloured solid line in Fig. 3b). This strongly supports the hypothesis that the Hahn-echo coherence time is limited by electrical noise. As already argued before, LSES_G1_ and LSES_G2_ indeed quantify the susceptibility of the hole spin to electric field fluctuations parallel and perpendicular to the channel, respectively.

The best fit in Fig. 3b is obtained with ${S}_{{{{\rm{G}}}}1}^{{{{\rm{hf}}}}}={(1.7\,\upmu {{{\rm{V}}}}/\sqrt{{{{\rm{Hz}}}}})}^{2}$ and ${S}_{{{{\rm{G}}}}2}^{{{{\rm{hf}}}}}={(66\,{{{\rm{nV}}}}/\sqrt{{{{\rm{Hz}}}}})}^{2}$. We speculate that the large ${S}_{{{{\rm{G}}}}1}^{{{{\rm{hf}}}}}/{S}_{{{{\rm{G}}}}2}^{{{{\rm{hf}}}}}$ ratio results from an artificial enhancement of ${S}_{{{{\rm{G}}}}1}^{{{{\rm{hf}}}}}$ accounting for hidden sources of electric field fluctuations along the silicon nanowire. Certainly, equation (1) misses the contribution from the electrical noise on G3, the LSES of which could not be measured. For reasons of symmetry, we expect LSES_G3_ to be comparable to LSES_G1_. A possible additional source of longitudinal electric field fluctuations are the randomly oscillating charges and dipoles in the silicon nitride spacers between the gates. Because these noise sources are closer to QD2 than is gate G1, and because they are much less screened by the hole gas beneath, they presumably make a large contribution to the apparent ${S}_{{{{\rm{G}}}}1}^{{{{\rm{hf}}}}}$ when lumped into ∝LSES_G1_ terms.

To further investigate the hole spin coherence, we implement Carr–Purcell–Meiboom–Gill (CPMG) sequences at the most favourable field orientation *θ*_*zx*_ = 99^∘^. These consist in increasing the number of π pulses cancelling faster and faster dephasing mechanisms. Figure 3c displays the CPMG echo amplitudes as a function of the total waiting time *τ*_wait_ for series of *N*_π_ = 2^*n*^π pulses, where *n* is an integer ranging from 1 to 8. The CPMG decay times ${T}_{2}^{{{{\rm{CPMG}}}}}$ extracted from Fig. 3c (see caption) are plotted against *N*_π_ in Fig. 3d. As expected, the data points follow a power law ${T}_{2}^{{{{\rm{CPMG}}}}}\propto {N}_{\uppi }^{\gamma }$, where $\gamma =\frac{\alpha }{\alpha +1}$ for a ∝1/*f*^*α*^ noise spectrum^4^. The best-fit value *γ* = 0.34 yields again *α* ≈ 0.5. For the largest sequence of 256 π pulses, we find ${T}_{2}^{{{{\rm{CPMG}}}}}=0.4\,{\mathrm{ms}}$, an exceptionally long coherence for a hole spin^34^.

**Fig. 4:**
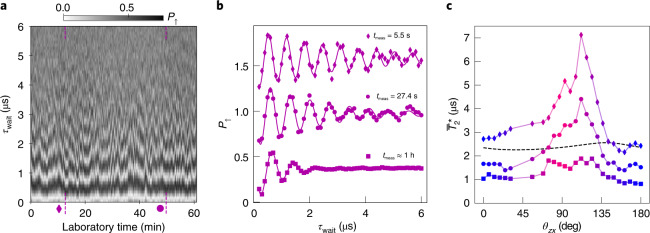
Free induction decay. **a**, Collection of 600 Ramsey oscillations as a function of *τ*_wait_, the free evolution time between two π_*x*_/2 pulses, at *θ*_*zx*_ = 118^∘^. The applied microwave frequency is detuned by ~700 kHz from the Larmor frequency. Each Ramsey oscillation is measured in ∼5.5 s. The locations of the representative traces shown in **b** are indicated by a diamond and a dot. **b**, Selected averages of Ramsey oscillations taken over different measurement times: *t*_meas_ = 5.5 s corresponding to a single trace (diamonds); *t*_meas_ = 27.5 s, corresponding to five consecutive traces (circles); *t*_meas_ ≈ 1 h, corresponding to the full set of 600 traces (squares). The solid lines are fits to Gaussian decaying oscillations. Note that the decay time ${T}_{2}^{* }$ depends on the chosen subset of consecutive traces (except for *t*_meas_ ≈ 1 h), which is a signature of non-ergodicity^36^ at small *t*_meas_ (Supplementary Information, section 4). Hence we observe a distribution of ${T}_{2}^{* }$ values with mean ${\overline{T}}_{2}^{* }$. **c**, Mean ${\overline{T}}_{2}^{* }$ for different *t*_meas_ (same symbols as in **b**) as a function of the magnetic field angle *θ*_*zx*_. The solid lines are guides to the eye. The dashed black line is the calculated dephasing time due to hyperfine interactions (Supplementary Information, section 5).

Finally, to gain insight into the low-frequency noise acting on the hole spin, we perform systematic measurements of the inhomogeneous dephasing time ${T}_{2}^{* }$. To this aim, we apply Ramsey control sequences consisting of two π/2 pulses separated by a variable delay *τ*_wait_. Contrary to Hahn-echo, the dephasing induced by low-frequency noise sources is not cancelled due to the absence of the refocusing π pulse. Figure 4a displays *P*_↑_ for a series of identical Ramsey sequences recorded on an overall time frame of 1 h, with each sequence lasting approximately 5.5 s. The next step is to average *P*_↑_(*τ*_wait_) on a subset of consecutive sequences measured within a total time *t*_meas_. This way, an averaged Ramsey oscillation is obtained for each *t*_meas_, the amplitude of which is fitted to a Gaussian-decay function yielding ${T}_{2}^{* }({t}_{{{{\rm{meas}}}}})$. Representative Ramsey data sets and corresponding fits are shown in Fig. 4b for three values of *t*_meas_. The inhomogeneous dephasing time decreases with increasing *t*_meas_ due to the contribution of noise components with lower and lower frequency. To unveil the angular dependence of ${T}_{2}^{* }$, we repeat the same measurement for different magnetic field orientations. The results are plotted in Fig. 4c for the same three values of *t*_meas_. The overall anisotropy of the Hahn-echo decay time of Fig. 3b can still be identified, although it reduces at large *t*_meas_ starting from *t*_meas_ > 50 s.

**Extended Data Fig. 6:**
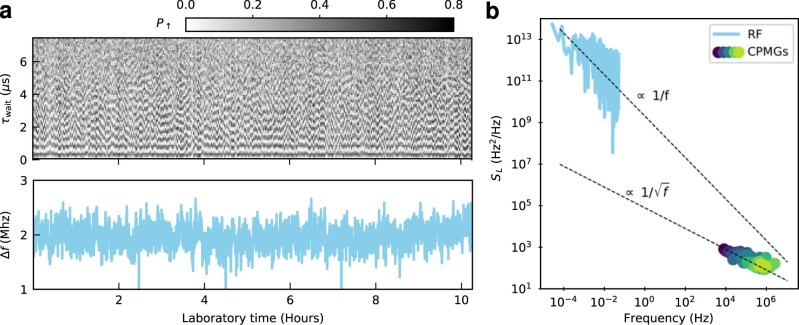
Noise spectrum. **(a)** (top) Ramsey fringes as a function of *τ*_w*ait*_ acquired during 10 hours, at *θ*_*zx*_ = 90^∘^. Each fringe oscillates at the frequency Δ*f* = *f*_M*W*1_ − *f*_L_. A single fringe takes roughly 10 s to record. (bottom) Δ*f*, obtained via Fourier transform of the Ramsey fringes, versus laboratory time. **(b)** Power spectral density of the noise on the Larmor frequency. The low-frequency spectrum (RF) is calculated from (a) and is roughly proportional to 1/*f*, as outlined by the upper dashed line. The high frequency spectrum (colored dots) is extracted from CPMG measurements with *N*_*π*_ from 2 to 256, and is proportional to 1/*f*^0.5^ (lower dashed line).

However, if the 1/*f*^0.5^ charge noise prevailed over the whole mHz to MHz range, ${T}_{2}^{* }$ would be ∼50 μs when ${T}_{2}^{{{{\rm{E}}}}}\approx 88\,\upmu{\mathrm{s}}$ (Supplementary Information, section 3), well above the 7 μs seen in Fig. 4c. The power spectrum *S*(*f*) at low frequency can be extracted from the data of Fig. 4a (Extended Data Fig. 6). This reveals a 1/*f*^*α*^ noise with *α* closer to 1, and a power (at 1 Hz) four orders of magnitude larger than the one expected by extrapolating the high-frequency 1/*f*^0.5^ noise inferred from CPMG. The change of exponent *α* and amplitude of *S*(*f*) when going from the mHz to the MHz points to the presence of different mechanisms dominating the dephasing at low and high frequencies. We note that the ${T}_{2}^{* }\approx 1{\text{--}}2\,\upmu{\mathrm{s}}$ measured at long *t*_meas_ is below but fairly close to the expected hole spin dephasing time due to hyperfine interactions with the naturally present ^29^Si nuclear spins^25^ (see the dashed line in Fig. 4c, and Supplementary Information, section 5 for details). This suggests that low-frequency dephasing may be partially due to such hyperfine interactions.

## Conclusions

We report on a spin qubit with electrical control and single-shot readout based on a single hole in a silicon nanowire device issued from an industrial-grade fabrication line. The hole wave function and corresponding *g*-factors could be modelled with an excellent level of accuracy in these types of devices, denoting a relatively low level of structural and charge disorder. The hole-spin coherence was found to be limited by a 1/*f*^0.5^ charge noise at high frequencies (10^4^−10^6^ Hz), with a strong dependence on the magnetic-field orientation that could be faithfully accounted for by the spin-electric susceptibilities. A largely enhanced spin coherence was measured at the sweet-spot angle, far beyond the current state-of-the-art for hole-spin qubits and close to the best figures reported for ^28^Si electron-spin qubits electrically driven via a micromagnet. Our study of the inhomogeneous dephasing time revealed a much stronger noise at low frequencies (10^−4^−10^−2^ Hz) that could be partially ascribed to the expected hyperfine interaction. In this scenario, the possible introduction of isotopically purified silicon devices would lead to a significant improvement of hole–spin coherence in the low-frequency range. Finally, we would like to emphasize that such sweet spots should be ubiquitous in hole spin qubit devices^21^, and that a careful design and choice of operation point can make them usefully robust to disorder (see example in Supplementary Information, section 1). The engineering of sweet spots should therefore open new opportunities for an efficient realization of multi-qubit or coupled spin-photon systems^35^.

## Methods

### Device

The device is a four-gate silicon-on-insulator nanowire transistor fabricated in an industry-standard 300 mm CMOS platform^11^. The undoped [110]-oriented silicon nanowire channel is 17 nm thick and 100 nm wide. It is connected to wider boron-doped source and drain pads used as reservoirs of holes. The four wrapping gates (G1–G4) are 40 nm long and are spaced by 40 nm. The gaps between adjacent gates and between the outer gates and the doped contacts are filled with silicon nitride (Si_3_N_4_) spacers. The gate stack consists of a 6-nm-thick SiO_2_ dielectric layer followed by a metallic bilayer with 6 nm of TiN and 50 nm of heavily doped polysilicon. The yield of the four-gate devices across the full 300 mm wafer reaches 90% and their room temperature characteristics exhibit excellent uniformity (see Supplementary Information, section 6 for details).

### Dispersive readout

**Extended Data Fig. 7:**
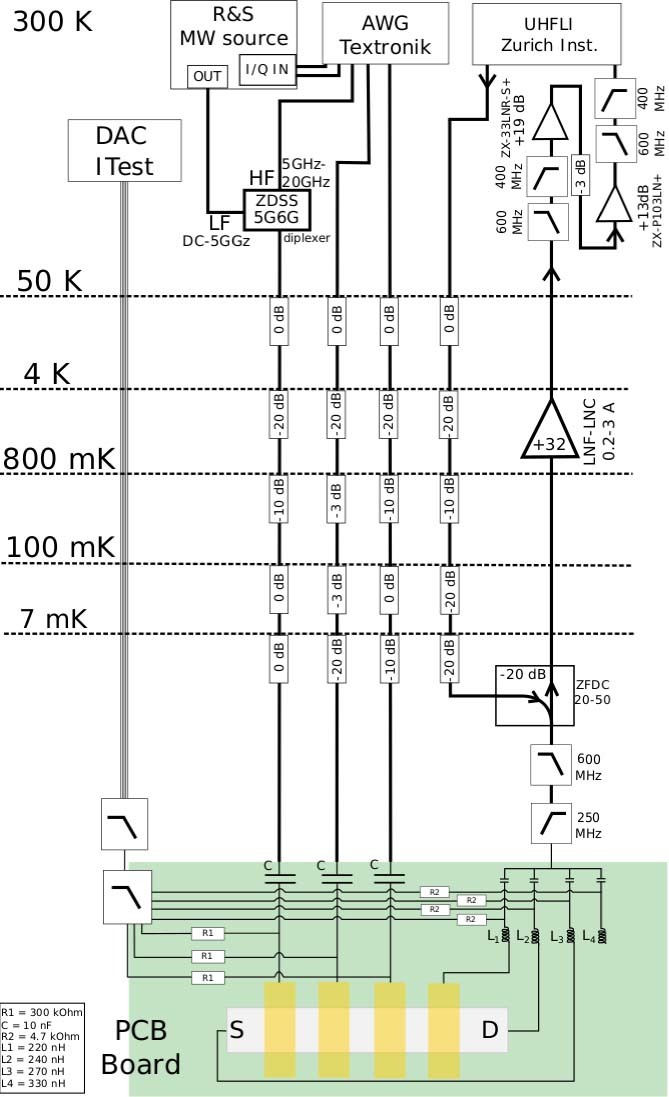
Experimental set-up. Dilution fridge with all electrical connections to the sample. We operate in a dilution refrigerator system equipped with a three-axis vector superconducting magnet. The main solenoid magnet produces a magnetic field of up to 6 T in the *z* direction, while both transverse Helmholtz coils ramp up to 1 T in the *x* and *y* directions. However, one of the axis was broken during the experiment. Therefore, after recording Fig. 1d of the main text, the sample was warmed up, physically rotated by 90^∘^, and cooled down again to record Fig. 1e. 24 twisted pairs are filtered at the mixing chamber by 6 low pass filters. The DC gate voltages are generated by Itest high stability voltage sources (BE2141). To perform charge and spin manipulation, semi-rigid coaxial lines with 20 GHz bandwidth are routed to G1, G2 and G3 using on-PCB bias tees. Microwave frequency signals are supplied by a vector signal generator (R&S SMW200A) with IQ modulating signals originating from two channels of an arbitrary waveform generator (AWG) Tektronix AWG5200. Other channels of the AWG are used to generate the pulse sequences. The homodyne readout of the resonator connected to the drain electrode is performed with a Zurich Instrument UHFLI lock-in with an excitation power of − 105 dBm at the PCB stage. The reflected signal from the resonator is amplified at 4 K with an ultra-low noise cryogenic amplifier LNF-LNC0.2-3A.

Similar to charge detection methods recently applied to silicon-on-insulator nanowire devices^37,38^, we accumulate a large hole island under gates G3 and G4, as sketched in Fig. 1a. The island acts both as a charge reservoir and electrometer for the quantum dot QD2 located under G2. However, unlike the aformentioned earlier implementations, the electrometer is sensed by radiofrequency dispersive reflectometry on a lumped element resonator connected to the drain rather than to a gate electrode. To this aim, a commercial surface-mount inductor (*L* = 240 nH) is wire bonded to the drain pad (see Extended Data Fig. 7 for the measurement set-up). This configuration involves a parasitic capacitance to ground *C*_p_ = 0.54 pF, leading to resonance frequency *f* = 449.81 MHz. The high value of the loaded quality factor *Q* ≈ 10^3^ enables fast, high-fidelity charge sensing. We estimate a charge readout fidelity of 99.6% in 5 μs, which is close to the state-of-the-art for silicon MOS devices^39^. The resonator characteristic frequency experiences a shift at each Coulomb resonance of the hole island, that is, when the electrochemical potential of the island lines up with the drain Fermi energy. This leads to a dispersive shift in the phase *ϕ*_drain_ of the reflected radiofrequency signal, which is measured through homodyne detection.

### Energy-selective single-shot readout of the spin state of the first hole in QD2

Extended Data Fig. 1a displays the stability diagram of the device as a function of *V*_G2_ and *V*_G3_ when a large quantum dot (acting as a charge sensor) is accumulated under gates G3 and G4. The dashed grey lines outline the charging events in the quantum dot QD2 under G2, detected as discontinuities in the Coulomb peak stripes of the sensor dot. The lever-arm parameter of gate G2 is *α*_G2_ ≈ 0.37 eV V^−1^, as inferred from temperature-dependence measurements. Comparatively, the lever-arm parameter of gate G1 with respect to the first hole under G2, *α*_G1_ ≈ 0.03 eV V^−1^, is much smaller. The charging energy, measured as the splitting between the first two charges, is *U* = 22 meV. Extended Data Fig. 1b shows a zoom on the stability diagram around the working point used for single-shot spin readout in the main text. The three points labelled Empty (E), Load (L) and Measure (M) are the successive stages of the readout sequence sketched in Extended Data Fig. 1c. The quantum dot is initially emptied (E) before loading (L) a hole with a random spin. Both spin states are separated by the Zeeman energy *E*_Z_ = *gμ*_B_*B* where *g* is the *g*-factor, *μ*_B_ is the Bohr magneton and *B* is the amplitude of the magnetic field. This opens a narrow window for energy-selective readout using spin to charge conversion^40^. Namely, we align at stage M the centre of the Zeeman split energy levels in QD2 with the chemical potential of the sensor. In this configuration, only the excited spin-up hole can tunnel out of QD2 while only spin-down holes from the sensor can tunnel in. These tunnelling events are detected by thresholding the phase of the reflectometry signal of the sensor to achieve single-shot readout of the spin state. Typical time traces of the reflected signal phase at stage M, representative of a spin up (spin down) in QD2, are shown in Extended Data Fig. 1d. We used this three-stage pulse sequence to optimize the readout. For that purpose, the tunnel rates between QD2 and the charge sensor were adjusted by fine tuning *V*_G3_ and *V*_G4_. For the spin-manipulation experiment discussed in the main text, we use a simplified two-stage sequence for readout by removing the empty stage. The measure stage duration is set to 200 μs for all experiments, while the load stage duration (seen as a manipulation stage duration) ranges from 50 μs to 1 ms. To obtain the spin-up probability *P*_↑_ after a given spin manipulation sequence, we repeat the single-shot readout a large number of times, typically 100–1,000 times.

### Pulse sequences

For Ramsey, Hahn-echo, phase-gate and CPMG pulse sequences, we set a π/2 rotation time of 50 ns. Given the angular dependence of *F*_Rabi_, we calibrate the microwave power required for this operation time for each magnetic field orientation. We also calibrate the amplitude of the π pulses to achieve a π rotation in 150 ns. In extracting the noise exponent *γ* from CPMG measurements, we do not include the time spent in the π pulses (this time amounts to about 10% of the duration of each pulse sequence).

### Noise spectrum

We measured 3,700 Ramsey fringes over *t*_tot_ = 10.26 h. For each realization, we varied the free evolution time *τ*_wait_ up to 7 μs, and averaged 200 single-shot spin measurements to obtain *P*_↑_ (Extended Data Fig. 6a, top). The fringes oscillate at the detuning Δ*f* = ∣*f*_MW1_ − *f*_L_∣ between the MW1 frequency *f*_MW1_ and the spin resonance frequency *f*_L_. To track low-frequency noise on *f*_L_, we make a Fourier transform of each fringe and extract its fundamental frequency Δ*f* reported in Extended Data Fig. 6a (bottom). Throughout the experiment, *f*_MW1_ is set to 17 GHz. The low-frequency spectral noise on the Larmor frequency (in units of Hz^2^ Hz^−1^) is calculated (here we make use of two-sided power spectral densities, which are even with respect to the frequency) from Δ*f*(*t*) as^4^:

2 $${S}_{\mathrm{L}}=\frac{{t}_{{{{\rm{tot}}}}}{\left|{{{\rm{FFT}}}}[{{\Delta }}f]\right|}^{2}}{{N}^{2}}\,,$$

where FFT[Δ*f*] is the fast Fourier transform (FFT) of Δ*f*(*t*) and *N* is the number of sampling points. We observe that the low-frequency noise, plotted in Extended Data Fig. 6b, behaves approximately as *S*_L_(*f*) = *S*^lf^(*f*_0_/*f*) with *S*^lf^ = 10^9^ Hz^2^ Hz^−1^, which is comparable to what has been measured for a hole spin in natural germanium^41^. To further characterize the noise spectrum, we add the CPMG measurements as coloured dots in Extended Data Fig. 6b^4^:

3 $${S}_{\mathrm{L}}\left({N}_{\uppi }/(2{\tau }_{{{{\rm{wait}}}}})\right)=-\frac{\ln ({A}_{{{{\rm{CPMG}}}}})}{2{\uppi }^{2}{\tau }_{{{{\rm{wait}}}}}},$$

where *A*_CPMG_ is the normalized CPMG amplitude. As discussed in the main text, the resulting high-frequency noise scales as ${S}^{{{{\rm{hf}}}}}{({f}_{0}/f)}^{0.5}$, where *S*^hf^ = 8 × 10^4^ Hz^2^ Hz^−1^ is four orders of magnitude lower than *S*^lf^. This high-frequency noise appears to be dominated by electrical fluctuations, as supported by the correlations between the Hahn-echo/CPMG *T*_2_ and the LSESs. Additional quasi-static contributions thus emerge at low frequency, and may include hyperfine interactions (Supplementary Information, section 5).

### Modelling

The hole wave functions and *g*-factors are calculated with a six-band **k** ⋅ **p** model^26^. The screening by the hole gases under gates G1, G3 and G4 is accounted for in the Thomas–Fermi approximation. As discussed extensively in Supplementary Information, section 1, the best agreement with the experimental data is achieved by introducing a moderate amount of charge disorder. The theoretical data displayed in Figs. 1, 2 and Extended Data Fig. 3 correspond to a particular realization of this charge disorder (point-like positive charges with density *σ* = 5 × 10^10^ cm^−2^ at the Si/SiO_2_ interface and *ρ* = 5 × 10^17 ^cm^−3^ in bulk Si_3_N_4_). The resulting variability, and the robustness of the operation sweet spots with respect to disorder, are discussed in Supplementary Information, section 1. The rotation of the principal axes of the *g*-tensor visible in Fig. 1d,e are most probably due to small inhomogeneous strains (<0.1%); however, in the absence of quantitative strain measurements, we have simply shifted *θ*_*zx*_ by ∼−25° and *θ*_*zy*_ by ∼10° in the calculations of Figs. 1, 2 and Extended Data Fig. 3.

## Online content

Any methods, additional references, Nature Research reporting summaries, source data, extended data, supplementary information, acknowledgements, peer review information; details of author contributions and competing interests; and statements of data and code availability are available at 10.1038/s41565-022-01196-z.

## Supplementary information

Supplementary Information Supplementary sections 1–6.

## Data Availability

All of the data used to produce the figures in this paper and to support our analysis and conclusions are available at https://zenodo.org/search?page=1&size=20&q=6638442. This repository includes the original data, jupyter notebooks for data analysis and figure plotting. Additional data are available upon reasonable request to the corresponding author.

## Code availability

The code is part of the Commisariat à l’Energie Atomique et aux Energies Alternatives strategy and could not be made public. However, the authors are ready to collaborate with anyone interested in the modelling tools used in this work, as they already do with several international teams.
