# Supplementary material for: A single hole spin with enhanced coherence in natural silicon
Source: Nat Nanotechnol. 2022 Sep 22;17(10):1072–7. doi: 10.1038/s41565-022-01196-z (PMC9576591; doi:10.1038/s41565-022-01196-z)
Supplement: Supplementary file 1 — Supplementary sections 1–6. [file 41565_2022_1196_MOESM1_ESM.pdf]

nature portfolio

https://doi.org/10.1038/s41565-022-01196-z

Supplementary information

A single hole spin with enhanced coherence in natural silicon

In the format provided by the authors and unedited

Supplementary information for "A single hole spin with enhanced coherence in natural silicon"

N. Piot,[1,*] B. Brun,[1,*] V. Schmitt,[1] S. Zihlmann,[1] V. P. Michal,[2] A. Apra,[1] J. C. Abadillo-Uriel,[2] X. Jehl,[1] B. Bertrand,[3] H. Niebojewski,[3] L. Hutin,[3] M. Vinet,[3] M. Urdampilleta,[4] T. Meunier,[4] Y.-M. Niquet,[2] R. Maurand,[1,†] and S. De Franceschi[1,‡]

[1] *Univ. Grenoble Alpes, CEA, Grenoble INP, IRIG-Pheliqs, Grenoble, France.*
[2] *Univ. Grenoble Alpes, CEA, IRIG-MEM-L_Sim, Grenoble, France.*
[3] *Univ. Grenoble Alpes, CEA, LETI, Minatec Campus, Grenoble, France.*
[4] *Univ. Grenoble Alpes, CNRS, Grenoble INP, Institut Néel, Grenoble, France.*
(Dated: June 13, 2022)

CONTENTS

S1. Modeling of the g-factors 1
A. Methodology 1
B. Discussion 2
1. Nature of the hole states. 2
2. Enhancement of the lateral electric field by disorder. 3
3. Strains and the rotations of the principal magnetic axes. 4
C. Outlook: Robustness of the sweet spots with respect to disorder 5

S2. Rabi oscillations at the sweet spot 7

S3. Pure dephasing with uncorrelated noise sources 8
A. Free induction decay 9
B. Hahn Echo sequence 9
C. CPMG sequence 10

S4. T_2^* in the non ergodic regime 10

S5. Hyperfine interaction limit for the inhomogeneous dephasing time 11

S6. Uniformity and quality of the samples at the wafer scale 12

References 13

S1. MODELING OF THE g-FACTORS

In this section, we give an overview of the methodology used to model the device, then discuss the outcome of the simulations and the comparison with experimental data. We finally provide arguments on the robustness of the sweet spots as an outlook.

A. Methodology

The device (Extended Data Fig. 2a) is modeled as a [110]-oriented rectangular nanowire channel with width $W = 100$ nm and height $H = 17$ nm lying on a 145 nm thick buried oxide (BOX). Four 40 nm long and 50 nm tall front gates, separated by 40 nm long Si_3N_4 spacers, are laid across the channel. They are insulated by a 6 nm thick SiO_2 layer.

* Contributed equally to the work.
† romain.maurand@cea.fr
‡ silvano.defranceschi@cea.fr

Highly doped source and drain reservoirs ($N_{\mathrm{A}} = 10^{20}$ cm^{-3}) are overgrown at both ends of the channel. The whole device is embedded in a 35 nm thick $\mathrm{Si_3N_4}$ contact etch stop layer (CESL), and coated with a $\simeq 250$ nm thick oxide. The silicon substrate beneath can be used as a back gate, and a wide metal line above (at the Metal 1 level) as an extra top gate. These top and back gates, as well as the source and drain are grounded in the simulations.

The potential landscape $V(\mathbf{r})$ in the device is first computed with a finite volumes Poisson solver [1]. Screening by the holes accumulated in the source, drain and below the gates G1, G3, and G4 is accounted for in the Thomas-Fermi approximation. Namely, these accumulations are modeled as locally homogeneous 3D hole gases, with density:

$$p(\mathbf{r}) = N_v F_{1/2}\left[\beta\left(E_v - eV(\mathbf{r}) - \mu\right)\right] \,, \tag{1}$$

where $F_{1/2}$ is a Fermi-Dirac integral, $N_v = (3.5\times10^{15}\,\mathrm{cm}^{-3}.\mathrm{K}^{-3/2})T^{3/2}$ is the effective density of states in the valence band, $E_v - \mu$ is difference between the valence band edge energy and the chemical potential (chosen to match the threshold voltage of the device), and $\beta = 1/k_BT$ with T the temperature. This equation is solved self-consistently together with Poisson's equation:

$$\varepsilon_0 \nabla\varepsilon_r(\mathbf{r})\cdot\nabla V(\mathbf{r}) = -e\left[p(\mathbf{r}) + \rho_{\mathrm{test}}(\mathbf{r}) + \rho_{\mathrm{trap}}(\mathbf{r}) - N_{\mathrm{A}}(\mathbf{r})\right] \,, \tag{2}$$

where $\varepsilon_0\varepsilon_r(\mathbf{r})$ is the material-dependent dielectric constant, $\rho_{\mathrm{test}}(\mathbf{r})$ is a test charge distribution that mimics a single hole within the dot QD2 under G2, and $\rho_{\mathrm{trap}}(\mathbf{r})$ is a distribution of charge traps used to assess the effects of disorder. The test charge $\rho_{\mathrm{test}}(\mathbf{r})$ prevents the Thomas-Fermi density from flooding the dot, as this approximation is notoriously inaccurate in the few holes regime. The bias voltages are used to set the boundary conditions on the gates.

The test charge distribution $\rho_{\mathrm{test}}(\mathbf{r})$ is practically modeled as a homogeneous ellipsoid with total charge $+1$, centered on the average position $\mathbf{R} = (\langle x\rangle, \langle y\rangle, \langle z\rangle)$ of the hole (computed *a posteriori* from the quantum-mechanical wave functions), with radii $a_x = \sqrt{3(\langle x^2\rangle - \langle x\rangle^2)}$, $a_y = \sqrt{3(\langle y^2\rangle - \langle y\rangle^2)}$, and $a_z = \sqrt{3(\langle z^2\rangle - \langle z\rangle^2)}$. As the potential $V_{\mathrm{QD}}(\mathbf{r})$ relevant for the Hamiltonian of the dot is that of the empty QD2, the self-consistent $V(\mathbf{r})$ is corrected from the contribution of $\rho_{\mathrm{test}}(\mathbf{r})$:

$$V_{\mathrm{QD}}(\mathbf{r}) = V(\mathbf{r}) - V_{\mathrm{test}}(\mathbf{r}) \,, \tag{3}$$

where $V_{\mathrm{test}}(\mathbf{r})$ is the potential created by $\rho_{\mathrm{test}}(\mathbf{r})$:

$$\varepsilon_0 \nabla\varepsilon_r(\mathbf{r})\cdot\nabla V_{\mathrm{test}}(\mathbf{r}) = -e\rho_{\mathrm{test}}(\mathbf{r}) \,. \tag{4}$$

As long as the dot and hole gases around remain sufficiently separated, the resulting $V_{\mathrm{QD}}(\mathbf{r})$ is only weakly dependent on the choice of $\rho_{\mathrm{test}}(\mathbf{r})$.

The wave functions in the potential $V_{\mathrm{QD}}(\mathbf{r})$ are then calculated on the same mesh with a finite differences 6 bands $\mathbf{k}\cdot\mathbf{p}$ model [1]. We use Luttinger parameters $\gamma_1 = 4.285$, $\gamma_2 = 0.339$, $\gamma_3 = 1.446$, split-off energy $\Delta = 44$ meV and Zeeman parameter $\kappa = -0.42$. The g-matrix of the ground-state is finally computed along the lines of Ref. [1]. The present formalism captures all the effects of spin-orbit coupling, including Rashba-type interactions when the dot moves along the channel [2, 3].

B. Discussion

In the following, we first discuss the nature of the hole states, and show that they tend to be confined in the top left or right corners of the channel by the lateral component of the electric field of the non-planar gate. We then argue why charge disorder needs to be introduced to reach lateral electric fields compatible with the experimental data. We discuss the resulting variability of the g-factors. Finally, we identify strain as the most likely mechanism for the rotation of the principal axes of the g-tensor evidenced on Fig. 1 of the main text.

1. *Nature of the hole states.*

If the device were "planar", the hole would be confined at the top (001) facet of the channel by the quasi-vertical electric field of gate G2. It would, therefore, show the fingerprints of an almost pure (001) heavy-hole, with a large $g_x \simeq -6\kappa + 2\gamma_h \simeq 4.84$, and much smaller g_y and g_z characteristic of the weak heavy-hole/light-hole mixing induced by the lateral confinement ($\gamma_h = 1.16$ being a correction that describes the heavy-hole/light-hole mixing by the magnetic vector potential) [3].

In our nanowire, non-planar geometry where each gate covers three facets of the nanowire, there is a significant in-plane electric field component that pushes the hole against the lateral $\{1\bar{1}0\}$ facets. This gives rise in principle to two symmetric "left" and "right" dots hybridized by tunneling across the channel. The calculated tunneling gap in such a large nanowire is, however, below 1 μeV in the present bias conditions. Therefore, any disorder that splits the left and right sides of the channel by more than a few μeV leads to the formation of two independent and non-degenerate "corner" dots with similar properties [4]. This is illustrated in Extended Data Fig. 2b-e, in a simpler setup with no hole gases under G1, G3 and G4. There we have added a positive charge on the left facet, which raises the energy of the left dot and break the degeneracy with the right dot; the calculated g-factors of the right dot are, however, little dependent on the exact position of the charge introduced on the left side of the channel. Note that there are no clear signatures of a second corner dot in the experimental data, probably because tunneling in and out of this dot occurs at an undetectable rate. As a matter of fact, tunnel rates can be highly sensitive to small perturbations of the potential landscape. Also note that it is practically impossible to determine whether the experimentally observed corner dot is actually on the left or on the right.

Given the width of the device, the hole is very responsive to the lateral electric field, and gets readily squeezed near one of the top corners of the channel, in a dot with comparable vertical and lateral extensions (Extended Data Fig. 2c,d). The enhancement of lateral with respect to vertical confinement admixes a light-hole envelope into the hole wave function, which results in a decrease of g_x ($\partial g_x/\partial V_{\mathrm{G2}} > 0$) and an increase of g_y and g_z ($\partial g_z/\partial V_{\mathrm{G2}} < 0$, see Extended Data Fig. 2b) [3, 5]. The mixing is particularly strong here because the structural vertical confinement is weak ($H = 17$ nm) so that the heavy-hole/light-hole gap is small. The g-factors (especially g_x and g_y) tend to saturate rapidly with increasingly negative V_{G2} as the heavily squeezed hole hardly responds any more to the vertical and lateral electric fields ($|\partial g_x/\partial V_{\mathrm{G2}}| \ll |\partial g_z/\partial V_{\mathrm{G2}}|$). The LSES computed at the purple star of Extended Data Fig. 2b are plotted as a function of θ_{zx} in Extended Data Fig. 2e. Interestingly, the zeros of $\mathrm{LSES}_{\mathrm{G2}}$ almost coincide with those of $\mathrm{LSES}_{\mathrm{G1}}$ and $\mathrm{LSES}_{\mathrm{G3}}$. Indeed, most electric field lines connect gate G2 to gates G1 and G3, so that the Larmor frequency of the hole is primarily a function of $V_{\mathrm{G2}} - (V_{\mathrm{G1}} + V_{\mathrm{G3}})/2$, and $\partial f_L/\partial V_{\mathrm{G1}} \approx \partial f_L/\partial V_{\mathrm{G3}} \approx -(\partial f_L/\partial V_{\mathrm{G2}})/2$.[1]

2. *Enhancement of the lateral electric field by disorder.*

Once screening by the holes gases under G1, G3 and G4 is accounted for, the lateral electric field is too weak to match the measured g-factors at the experimental bias point. This is highlighted in Extended Data Fig. 3a, where the symbols are the experimental g-factors and the dashed lines are the calculated ones. g_y remains actually smaller that g_x (at $\theta_{zx} = \theta_{zy} = 90°$). This discrepancy may result from inaccuracies in the Thomas-Fermi screening, and (more likely) from additional sources of localization such as disorder. In particular, holes in the channel may be captured by traps at the Si/SiO_2 interface (P_b defects) [6], and holes in the poly-silicon gates by traps in the Si_3N_4 spacers. Such positively charged traps repel the holes and tend to strengthen confinement in the corners, where the resulting potential is best screened by gate G2. The traps are introduced in the simulations as a random distribution of point charges at the Si/SiO_2 interface and in Si_3N_4. We can achieve similar g-factors with different combinations of P_b and bulk defects densities; the data displayed in the main text and in Extended Data Fig. 3a (solid lines) are computed for a particular realization of disorder with density $\sigma_{\mathrm{trap}} = 5 \times 10^{10}$ P_b defects/cm^2 at the Si/SiO_2 interface and density $\rho_{\mathrm{trap}} = 5 \times 10^{17}$ traps/cm^3 in Si_3N_4. This σ_{trap} is typical of Si/SiO_2 interface, while the chosen ρ_{trap} does not seem unrealistic given the known affinity of nitrides for charges [7]. The potential $V_{\mathrm{QD}}(\mathbf{r})$ and the single-hole wave function of this particular device are shown in Extended Data Fig. 3b,c. The distortions of the isopotential lines and wave function due to disorder are moderate but clearly visible. For the sake of completeness, interface roughness is also included in the simulations. It is characterized by rms fluctuations $\Delta = 0.3$ nm and correlation length $L_c = 8$ nm [6]. The model reproduces the main features of the experimental data, including the magnitude and anisotropy of the g-factors and $\mathrm{LSES}_{\mathrm{G2}}$ (Fig. 2 of the main text).

Choosing [001] as the quantization axis, the hole wave function of Extended Data Fig. 3b,c is a strong mixture of heavy ($\approx 54\%$ $|3/2, \pm 3/2\rangle_{[001]}$) and light ($\approx 43\%$ $|3/2, \pm 1/2\rangle_{[001]}$) envelopes (the reminder being a split-off component). Choosing instead $y = [1\bar{1}0]$ as the quantization axis, the hole appears as a majority $\approx 85\%$ $|3/2, \pm 3/2\rangle_{[1\bar{1}0]}$ envelope admixed with a minority $\approx 12\%$ $|3/2, \pm 1/2\rangle_{[1\bar{1}0]}$ component. The measured and computed $g_y > g_x$ is the salient fingerprint of the prevalence of $|3/2, \pm 3/2\rangle_{[1\bar{1}0]}$ over $|3/2, \pm 3/2\rangle_{[001]}$ components. The confinement being comparable along

---

[1] We emphasize that variations of V_{G1} do not only move the dot as a whole along the channel, but deform it on the way, which gives rise to the finite $\mathrm{LSES}_{\mathrm{G1}}$. Only joint, opposite variations $\delta V_{\mathrm{G1}} = -\delta V_{\mathrm{G3}}$ move the dot as a whole with negligible LSES, at least in the absence of hole gases under G1 and G3.

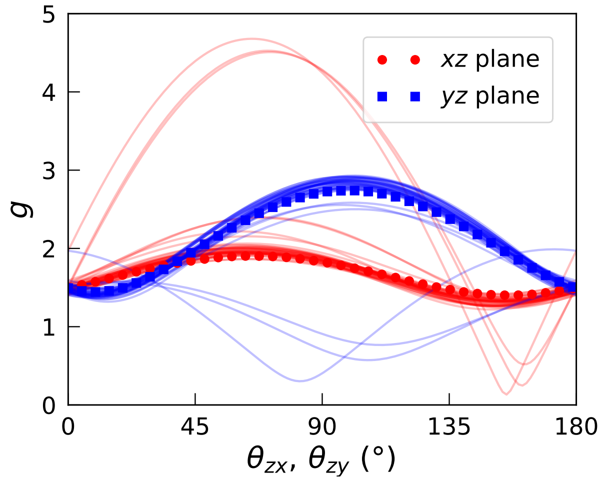

Figure S.1. **Variability of the calculated g-factors.** Same as Extended Data Fig. 3a; Each line is a different realization of the interface roughness and charge disorder. The interface roughness rms is $\Delta = 0.3$ nm and the correlation length is $L_c = 8$ nm [6]; The density of positively charged traps is $\sigma_{\rm trap} = 5 \times 10^{10}$ cm^{-2} at the Si/SiO$_2$ interface, and $\rho_{\rm trap} = 5 \times 10^{17}$ cm^{-3} in Si_3N_4.

x and y, the hole actually appears purest when quantized along $z = [110]$, where it stands as a $\approx 90\%\, |3/2, \pm 1/2\rangle_{[110]}$ envelope (as expected from $g_z < g_x, g_y$) [8].

The disorder gives rise to variability in the g-factors (dependence on the particular realization of the disorder [6]). This is outlined in Fig. S.1, which shows the g-factors calculated in 50 devices with different samples of disorder. Fourty-four out of the 50 devices still show g-factors in reasonable agreement with the experiment. Indeed, the g-factors tend to saturate once the hole is squeezed on a lateral facet as discussed above. In the 3 disorder configurations featuring large g_x and small g_y, the hole remains localized at the top interface because there are P_b defects near both corners.

Although we can reach a satisfactory agreement with the experimental $\mathrm{LSES_{G2}}$ for many realizations of the disorder, we systematically miss the anisotropy of $\mathrm{LSES_{G1}}$ (Fig. 2 of the main text). This discrepancy may result from limitations of our model. While we capture semi-quantitatively the strong screening of the electric field of gate G1 by the hole gas beneath ($|\partial f_L/\partial V_{\rm G1}| \ll |\partial f_L/\partial V_{\rm G2}|/2$, contrary to Extended Data Fig. 2e), the Thomas-Fermi approximation used to model this hole gas may not be accurate enough. It certainly misses quantization effects as well as the magnetic response of the hole gas. Given the large number of holes under gates G1/G3/G4, going beyond the Thomas-Fermi approximation is however far from trivial. Also, strain inhomogeneities when the dot is moved along the channel may play a role in $\mathrm{LSES_{G1}}$ (see below). The fact that $\mathrm{LSES_{G1}}$ is always negative can be explained by the presence of a charged P_b defect in the vicinity of gate G2 that pushes the dot towards gate G3, as shown in Extended Data Fig. 3c. The robustness of the LSES with respect to disorder will be further discussed in section S1 C.

3. Strains and the rotations of the principal magnetic axes.

We finally discuss the misalignment of the principal axes of the g-tensor with respect to the device axes. Indeed, the principal axes X, Y, Z of the calculated g-tensor are almost perfectly aligned with the device x, y and z axes, whereas those of the experimental g-tensor are slightly rotated [by $\approx 10°$ around x ($xyz \to xYz'$), then $\approx -25°$ around Y ($xYz' \to XYZ$)]. The large rotation around Y can hardly be accounted for by a simple misalignment of the sample. The fact that Z is not oriented along the channel implies a loss of the xy quasi-symmetry plane of gate G2 [1], and the existence of additional heavy-hole/light-hole mixing mechanisms. The disorder introduced in the previous section actually rotates the principal axes of the g-tensor, but by no more than a few degrees. The most likely scenario is that QD2 is slightly displaced towards G3 (as suggested above), and experiences small process and cool-down strains [1, 9]. In particular, shear strains control the phase of the heavy-hole/light-hole mixing matrix elements. In the basis

set and axes set of Ref. 3, they give rise to non-diagonal corrections to the g-matrix:

$$\delta g_{zy} \approx \frac{4\sqrt{3}\kappa d}{\Delta}\varepsilon_{yz},\ \delta g_{zx} \approx \frac{4\sqrt{3}\kappa d}{\Delta}\varepsilon_{xz},\ \delta g_{xy} = -\delta g_{yx} \approx -\frac{12\kappa b}{\Delta}\varepsilon_{xy}\,, \tag{5}$$

where $b = -2.1$ eV and $d = -4.85$ eV are the uniaxial and shear deformation potentials of the valence band of silicon, and Δ is the heavy-hole/light-hole gap. Therefore, the shear strains ε_{yz}, ε_{xz}, and ε_{xy} drive rotations of the principal magnetic axes around x, y and z respectively. Our simulations reproduce the experimental rotations assuming small $\varepsilon_{yz} \simeq 0.03\%$ and $\varepsilon_{xz} \simeq 0.08\%$, which highlights the sensitivity of such quantum devices to residual strains [1, 9, 10]. The assessment of the inherently inhomogeneous strains in such complex nanostructures remains, however, difficult (in particular in the nitrides), and goes beyond the scope of this work. In the absence of a complete distribution of strains (including hydrostatic and uniaxial components), we have practically shifted θ_{zx} by $\approx -25°$ and θ_{zy} by $\approx 10°$ in the simulations of Figs. 1 and 2 of the main text, and in Extended Data Fig. 3 and Fig.S.1, as if these rotations resulted from a misalignment of the sample with respect to the magnet axes. Note that the possible rotation of the principal axes around z can not be resolved since the g-factors have not been measured in the xy plane; yet it must be within $\pm 20°$ to reach a satisfactory agreement between theory and experiment.

Since uniaxial and shear strains rule the heavy-hole/light-hole mixing together with confinement, they can in principle help reduce the lateral confinement, hence the disorder needed to reach agreement with the experimental g-factors. We emphasize, though, that the dot becomes much more responsive to G2 once deconfined from the corner, so that $\partial f_L/\partial V_{\mathrm{G2}}$ increases significantly. Therefore, the magnitude of the experimental $\partial f_L/\partial V_{\mathrm{G2}}$, as well as the fact that the experimental g-factors match the saturation values calculated at large gate voltage/electric field (Extended Data Fig. 2b), support strong confinement in the corners and small strains.

To conclude, the present model captures and explains the most salient features of the experiment: the anisotropy of the g-factors, $g_y > g_x > g_z$, and of $\partial f_L/\partial V_{\mathrm{G2}}$ result from the balance between vertical and lateral confinement in the corner dot of a "thick" silicon film; $\partial f_L/\partial V_{\mathrm{G1}}$ is strongly screened by the hole gas accumulated under gate G1 and is, therefore, much smaller (in magnitude) than $\partial f_L/\partial V_{\mathrm{G2}}$. The remaining discrepancies (in particular the rotation of the principal axes of the g-tensor) are attributed to residual process and cool-down strains and to possible inaccuracies in the description of screening.

C. Outlook: Robustness of the sweet spots with respect to disorder

As an outlook, we investigate the robustness of the sweet spots evidenced in this work. For that purpose, we consider a more versatile "face-to-face" layout [11] where G2 is split into two independent gates $G_{2\mathrm{L}}$ and $G_{2\mathrm{R}}$ that overlap the left (L) and right (R) corners respectively (see Fig. S.2a). In this configuration, the corner dots are much more deterministic and stable since the potential on the left and right sides of the channel can be adjusted independently. To probe the robustness of possible sweet spots, we introduce positive charge traps at the surface of silicon with density $\sigma_{\mathrm{trap}} = 5 \times 10^{10}$ cm^{-2}.

We ground all gates except G2L ($V_{\mathrm{G2L}} = -50$ mV). In such a thick channel ($H = 20$ nm), the lateral electric field between gates G2R and G2L is already large enough to squeeze the dot on the lateral facet and reach the regime $g_y > g_x$ (alternatively, the back gate voltage can be made positive to strengthen confinement in the corners). We next compute the LSES with respect to the four gates (LSES$_{\mathrm{G1}}$, LSES$_{\mathrm{G2L}}$, LSES$_{\mathrm{G2R}}$ and LSES$_{\mathrm{G3}}$). The data collected for five representative configurations of disorder are plotted in Fig. S.2b,c for a magnetic field $\mathbf{B}$ in the xz and xy planes, respectively. In Fig. S.2c, θ_{xy} is the angle between the magnetic field $\mathbf{B}$ and the x axis. The two planes exhibit qualitative differences with respect to the variability of the LSES. In particular, the sweet spot in the xz plane is generally more sensitive to disorder (and even missing for one of the configurations). On the contrary, there is a remarkably robust sweet spot in the xy plane near $\theta_{xy} \approx 41°$.

The sweet spots in the xz and xy planes actually belong to the same "sweet line" running around the x axis [12]. The sweet spot is however more robust to disorder in the xy plane because in this class of devices $\partial g_x/\partial V \approx -\partial g_y/\partial V$ whatever the gate[2] – in other words, the in-plane electric field primarily shifts weight between g_x and g_y (also see Extended Data Fig. 2b). Therefore, for a given θ_{xy}, the derivative of the g-factor with respect to the gate voltage reads

$$\frac{\partial g}{\partial V} = \frac{\partial}{\partial V}\sqrt{g_x^2\cos^2\theta_{xy} + g_y^2\sin^2\theta_{xy}} = \frac{1}{g}\left(g_x\frac{\partial g_x}{\partial V}\cos^2\theta_{xy} + g_y\frac{\partial g_y}{\partial V}\sin^2\theta_{xy}\right) \approx \left(g_x\cos^2\theta_{xy} - g_y\sin^2\theta_{xy}\right)\frac{1}{g}\frac{\partial g_x}{\partial V}\,, \tag{6}$$

[2] In the language of Ref. 12, $\left|\frac{\partial \mathbf{g}}{\partial V}\cdot\mathbf{b}\right|$ is weakly dependent on the orientation $\mathbf{b} = (\cos\theta_{xy}, \sin\theta_{xy}, 0)$ of the magnetic field in the (xy) plane, with $\mathbf{g}$ the g-matrix.

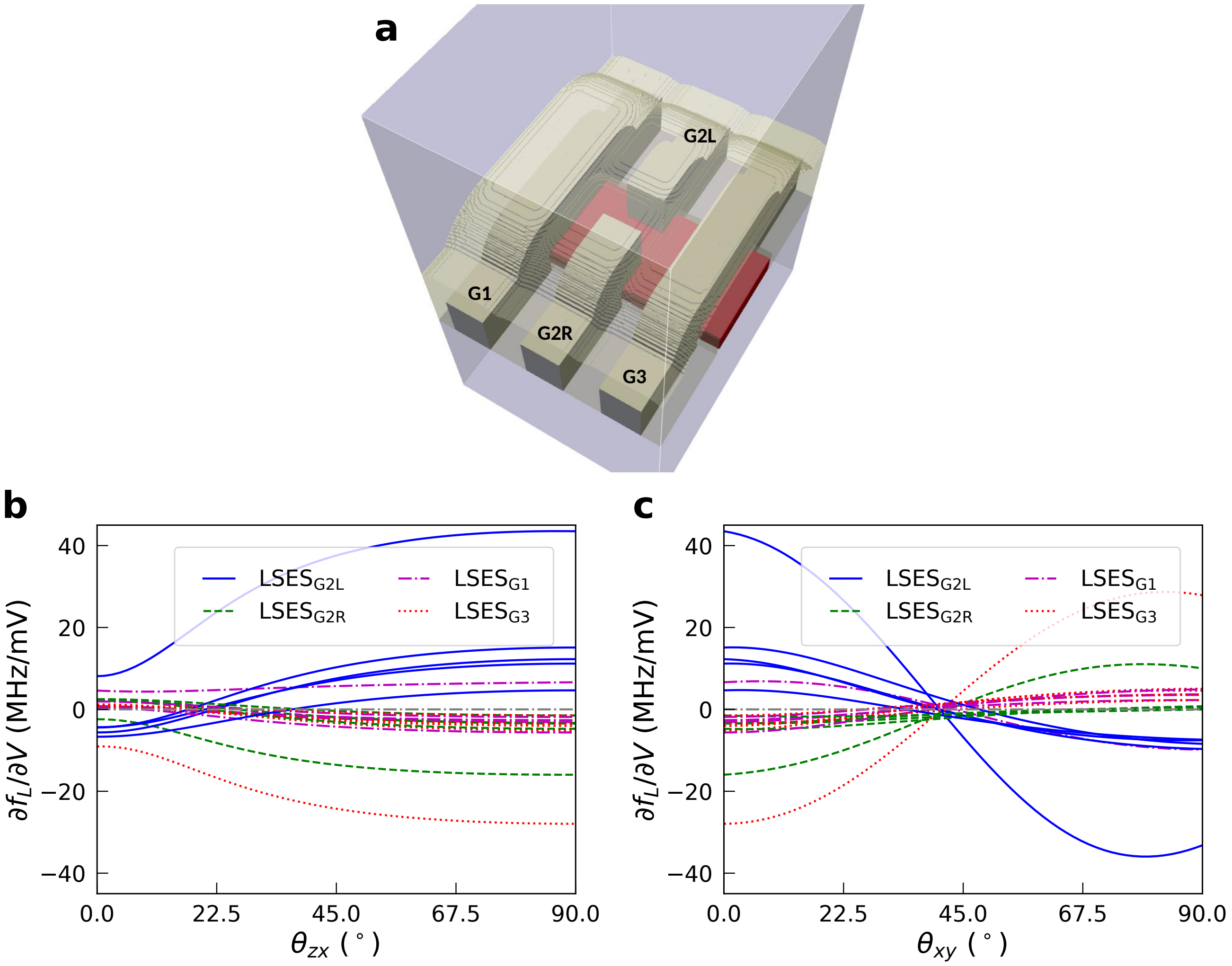

Figure S.2. **LSES of a face-to-face device.** **(a)** A face-to-face device similar to Extended Data Fig. 2a, but with G2 split into a left (G2L) and a right (G2R) gate separated by 40 nm. **(b)** $\mathrm{LSES_{G1}}$, $\mathrm{LSES_{G2L}}$, $\mathrm{LSES_{G2R}}$ and $\mathrm{LSES_{G3}}$ as a function of the angle θ_{zx} between the z axis and the magnetic field $\mathbf{B}$ in the xz plane, for five different random distributions of charge traps at the Si/SiO$_2$ interface ($\sigma_{\mathrm{trap}} = 5\times10^{10}$ cm^{-2}). The Larmor frequency is set to $f_L = 10$ GHz. **(c)** Same as (b) as a function of the angle θ_{xy} between the x axis and the magnetic field $\mathbf{B}$ in the xy plane.

which is zero when $\theta_{xy} \approx \frac{\pi}{2} \pm \arctan\sqrt{\frac{g_y}{g_x}}$. Hence, the position of the sweet spot is resilient to moderate disorder once g_x and g_y get close to saturation. Despite this saturation, the Rabi frequencies of the device of Fig. S.2 are still in the 10 MHz range for a 1 mV drive on gate G2L. They are actually maximal near the sweet spot in the xy plane [12] even in the presence of disorder.[3]

We expect such robust sweet spots to be ubiquitous in a large variety of silicon and germanium devices [12, 13]. Therefore, device optimization and improvements in material quality shall further enhance the performances of hole spin qubits in the near term.

---

[3] The sweet spot in the xy plane could not be probed with the present experimental setup, see Extended Data Fig. 7.

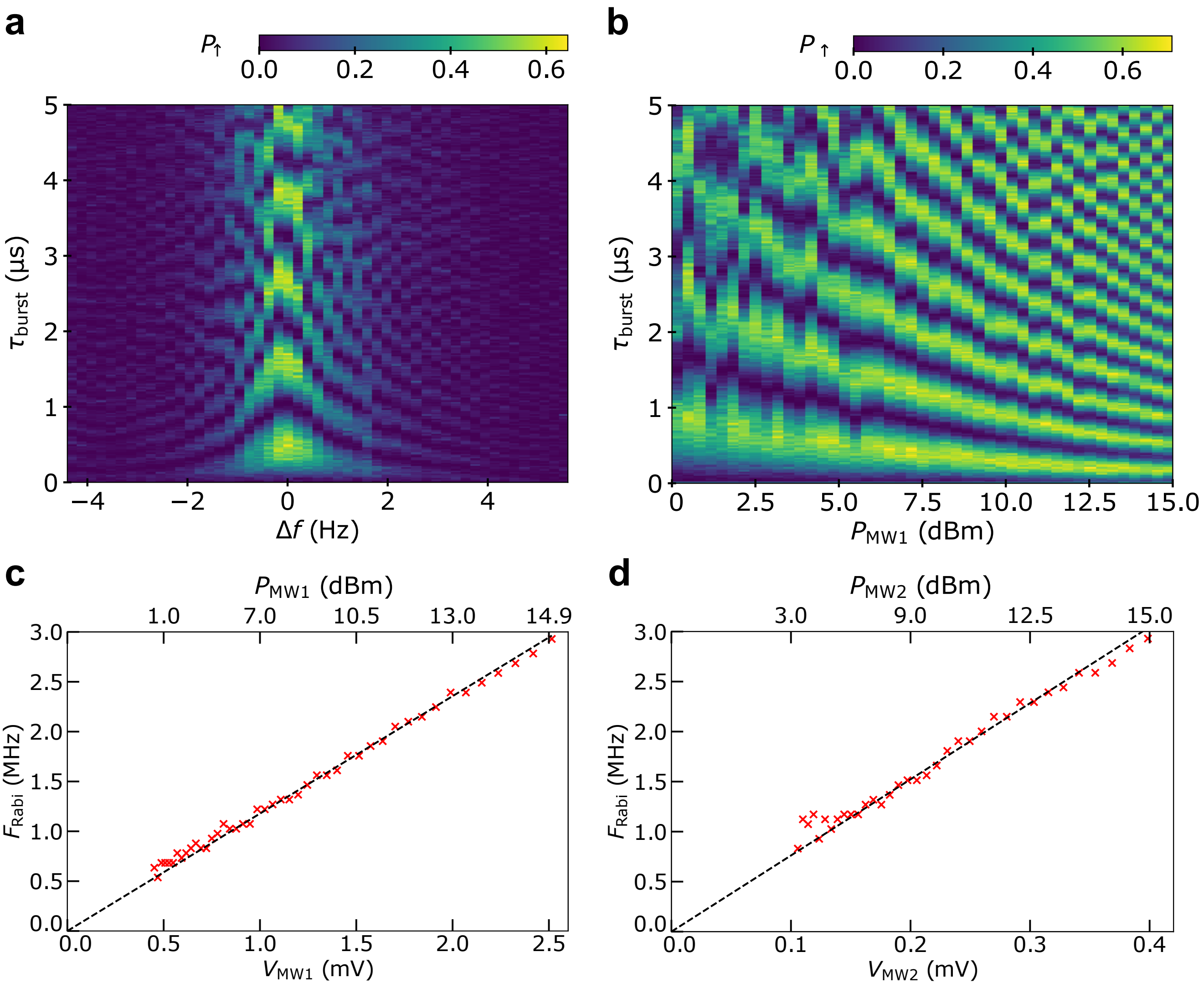

Figure S.3. **Electrical spin driving at the coherence sweet spot (a)** Chevron pattern at $\theta_{zx} = 99°$ recorded with the same pulse sequence as in Extended Data Fig. 4a. $P_\uparrow$ is plotted versus MW1 detuning from spin resonance ($\Delta f = f_{\text{MW1}} - f_L$) and MW1 burst duration τ_{burst}. The Larmor frequency is $f_L = 17\,\text{GHz}$ and the MW1 power on top of the fridge is $P_{\text{MW1}} = 5\,\text{dBm}$. **(b)** $P_\uparrow$ versus P_{MW1} and τ_{burst} for $\Delta f = 0$. **(c)** Rabi frequency extracted from (b) versus on chip MW1 amplitude V_{MW1} (symbols) assuming 30 dB attenuation from attenuators and 30 dB loss from cables at low temperature. The top axis is the power P_{MW1} delivered on top of the fridge. The dashed line is a linear fit with slope $F_{\text{Rabi}} = 1.2\,\text{MHz/mV}$, which evidences the absence of saturation at least up to 3 MHz. **(d)** Same as (c) but driving the spin using G2. The attenuation is larger on this line (46 dBm), so that the Rabi oscillations are actually 6 times faster on G2 (slope $F_{\text{Rabi}} = 7.6\,\text{MHz/mV}$) than on G1.

S2. RABI OSCILLATIONS AT THE SWEET SPOT

Sweet spots for coherence may result from a simple zero of the longitudinal spin-electric susceptibility, or from the complete decoupling of the hole from the electric field (for example if the wave function becomes centrosymmetric [1, 12]). In the latter case, Rabi oscillations (transverse spin-electric susceptibility) are also impossible. Figure S.3 demonstrates that the hole can still be manipulated electrically near the sweet spot for coherence at $\theta_{zx} = 99°$. In the experiment reported in the main text, the hole is driven by a microwave burst on gate G1. The Rabi frequency is found dependent on the magnetic field orientation (see Extended Data Fig. 5a), with a minimum around the sweet spot, where the hole spin still rotates up to $F_{\text{Rabi}} = 5$ MHz for an applied power of 20 dBm on top of the MW1 line. A microwave burst on gate G2 also enables spin rotation up to ~ 3 MHz at the sweet spot (see Fig. S.3d). However, we speculate that the Rabi frequency is only limited by the available microwave power and the line attenuation, since

we do not observe any saturation with increasing power. After conversion of the microwave power into gate voltage amplitudes, we find that the driving efficiency is much larger on gate G2 ($F_{\rm Rabi} = 7.6$ MHz/mV) than on gate G1 ($F_{\rm Rabi} = 1.2$ MHz/mV), which suggests that the spin could be rotated much faster by reducing the attenuation on the MW2 line.

S3. PURE DEPHASING WITH UNCORRELATED NOISE SOURCES

The hole can generally be described as an effective spin 1/2 with Hamiltonian [14]

$$H_s = \mathbf{S}\cdot\boldsymbol{\omega}_L(\mathbf{V}_{\rm G})\,. \tag{7}$$

Here $\mathbf{S} = \frac{\hbar}{2}\boldsymbol{\sigma}$ is the spin 1/2 operator and $\boldsymbol{\omega}_L(\mathbf{V}_{\rm G}) = \frac{\mu_B}{\hbar}\,\mathsf{g}(\mathbf{V}_{\rm G})\cdot\mathbf{B}$ stands for the spin precession (Larmor) vector, proportional to the product of the voltage-dependent g-tensor (or g-matrix [15]) $\mathsf{g}(\mathbf{V}_{\rm G})$ with the external magnetic field $\mathbf{B}$. $\mathbf{V}_{\rm G} = (V_{\rm G1}, V_{\rm G2}, \ldots, V_{{\rm G}n})$ is the set of voltages on gates G1, G2, ..., Gn. Each can be split into static and dynamical contributions $V_{{\rm G}i}(t) = V^0_{{\rm G}i} + \delta V_{{\rm G}i}(t)$, $\mathrm{V}^0_{{\rm G}i}$ being the bias voltage on gate Gi and $\delta V_{{\rm G}i}(t)$ the voltage noise responsible for qubit relaxation and decoherence.

The gate voltage noise introduces a random component $\delta\phi(t)$ to the qubit phase $\phi(t) = 2\pi f_L t + \delta\phi(t)$, where $f_L = \frac{\mu_B}{h}|\,\mathsf{g}(\mathbf{V}^0_{\rm G})\cdot\mathbf{B}|$ is the Larmor frequency. After free evolution over time t the accumulated random phase reads at first order in the noise [16, 17]:

$$\delta\phi(t) = 2\pi\int_0^t dt'\,\delta f_L(t') = 2\pi\int_0^t dt'\,\sum_i D_{{\rm G}i}\delta V_{{\rm G}i}(t')\,. \tag{8}$$

where $D_{{\rm G}i} = \partial f_L/\partial V^0_{{\rm G}i}$ is the LSES of gate Gi. More generally, for a dynamical decoupling pulse sequence the accumulated phase is [16–20]:

$$\delta\phi(t) = 2\pi\int_{-\infty}^{+\infty} dt'\,\sum_i D_{{\rm G}i}\delta V_{{\rm G}i}(t')\,\eta_t(t')\,, \tag{9}$$

where the function $\eta_t(t')$ describes the effects of the pulse sequence performed over time t. In particular, for free induction decay (Ramsey experiment),

$$\eta_t{}^{\rm R}(t') = \begin{cases} 1 & \text{if } 0 < t' < t, \\ 0 & \text{otherwise}\,, \end{cases} \tag{10}$$

and for a CPMG sequence with N_π π-pulses [21]:

$$\eta_t{}^{\rm CPMG}(t') = \sum_{k=0}^{N_\pi}(-1)^k\theta(t_{k+1}-t')\theta(t'-t_k)\,, \tag{11}$$

where θ is the Heaviside function, $t_k = (k-1/2)t/N_\pi$ for $k = 1, \ldots, N_\pi$, and by definition [21] $t_0 = 0$ and $t_{N_\pi+1} = t$. The Ramsey and the Hahn echo experiments are particular cases of the CPMG sequence with $N_\pi = 0$ and $N_\pi = 1$ respectively.

The dephasing experienced by the spin as a consequence of voltage noise is characterized by the decay of the off-diagonal element of the spin density matrix in the rotating frame [17]:

$$\langle\tilde\rho_{01}\rangle(t) = \tilde\rho_{01}(0)\langle e^{i\delta\phi(t)}\rangle = \tilde\rho_{01}(0)e^{-\frac{1}{2}\langle\delta\phi^2(t)\rangle}\,, \tag{12}$$

where $\langle\cdot\rangle$ denotes an ensemble average (over the random processes), and, for the general pulse sequence:

$$\langle\delta\phi^2(t)\rangle = 4\pi^2\int_{-\infty}^{+\infty} dt'\int_{-\infty}^{+\infty} dt''\,\sum_{i,j} D_{{\rm G}i}D_{{\rm G}_j}\langle\delta V_{{\rm G}i}(t')\delta V_{{\rm G}j}(t'')\rangle\,\eta_t(t')\,\eta_t(t'')\,. \tag{13}$$

Under the assumptions that the noise on the different gates are independent, and that their respective auto-correlation functions are homogeneous in time, we reach in frequency domain:

$$\langle\delta\phi^2(t)\rangle = 4\pi^2\int_{-\infty}^{+\infty} df\,\sum_i D^2_{{\rm G}i}S_{{\rm G}i}(f)\,|\tilde\eta_t(f)|^2\,, \tag{14}$$

where $S_{\mathrm{G}n}(f) = \int_{-\infty}^{+\infty} dt\, e^{-2i\pi ft}\, \langle \delta V_{\mathrm{G}n}(t)\delta V_{\mathrm{G}n}(0)\rangle$ is the Fourier transform of the auto-correlation function of the noise on gate Gn (the power spectrum according to the Wiener-Khinchin theorem), and $\tilde{\eta}_t(f) = \int_{-\infty}^{+\infty} dt\, e^{-2i\pi ft}\, \eta_t(t)$. Eq. (14) can also be formalized using the filter function concept [17, 21, 22]. We analyze below the different pulse sequences relevant for the present experiments.

A. Free induction decay

For the Ramsey sequence we have

$$\left|\tilde{\eta}_t{}^{\mathrm{R}}(f)\right|^2 = \left(\frac{\sin(\pi f t)}{\pi f}\right)^2 . \tag{15}$$

Therefore, $\left|\tilde{\eta}_t{}^{\mathrm{R}}(f)\right|^2/t^2$ is close to unity up to $|f| \sim 1/t \sim 1/T_2^*$, so that free induction decay is sensitive to noise in this whole range of frequencies. For low-frequency noise spectra of the form $S_{\mathrm{G}i}(f) = S_{\mathrm{G}i}^{\mathrm{lf}}\, f_0/\max(|f|, f_l)$ together with a (soft) high-frequency cutoff f_h, we get in the regime $2\pi f_l \ll 2\pi f_h \ll 1/t$:

$$\exp\left(-\frac{1}{2}\langle\delta\phi_R(t)^2\rangle\right) \approx \exp\left[-4\pi^2 t^2 \ln\left(\frac{f_h}{f_l}\right) f_0 \sum_i D_{\mathrm{G}i}^2\, S_{\mathrm{G}i}^{\mathrm{lf}}\right] \equiv \exp\left[-\left(\frac{t}{T_2^*}\right)^2\right] , \tag{16}$$

with [16]:

$$\frac{1}{T_2^*} \approx 2\pi \sqrt{\ln\left(\frac{f_h}{f_l}\right) f_0 \sum_i D_{\mathrm{G}i}^2\, S_{\mathrm{G}i}^{\mathrm{lf}}} . \tag{17}$$

As shown in the main text (Fig. 4), the averaged T_2^* decreases with increasing $t_{\mathrm{meas}} \sim 1/(2\pi f_{\mathrm{l}})$ as the experiment probes smaller and smaller noise frequencies.

We can also estimate the contribution of higher frequency noises with spectra $S_{\mathrm{G}i}(f) = S_{\mathrm{G}i}^{\mathrm{hf}} \sqrt{f_0/f}$. The Ramsey oscillations then decay as $\exp(-\frac{1}{2}\langle\delta\phi_R(t)^2\rangle) = \exp(-(t/T_{2,\mathrm{hf}}^*)^{3/2})$, where we define:

$$\frac{1}{T_{2,\mathrm{hf}}^*} = \left(\frac{16\pi^2}{3} f_0^{1/2} \sum_i D_{\mathrm{G}i}^2\, S_{\mathrm{G}i}^{\mathrm{hf}}\right)^{2/3} \approx 14 \left(f_0^{1/2} \sum_i D_{\mathrm{G}i}^2\, S_{\mathrm{G}i}^{\mathrm{hf}}\right)^{2/3} . \tag{18}$$

The low-frequency and high-frequency contributions to the decay of the Ramsey signal cross over at time $t_* = T_2^*(T_2^*/T_{2,\mathrm{hf}}^*)^3 \ll T_2^*$ when $T_2^* \ll T_{2,\mathrm{hf}}^*$, and the decay is dominated by the low-frequency noise when $t \gg t_*$.

B. Hahn Echo sequence

For the Hahn echo sequence,

$$\left|\tilde{\eta}_t{}^{\mathrm{E}}(f)\right|^2 = \frac{\sin^4(\pi f t/2)}{(\pi f/2)^2} . \tag{19}$$

Therefore, the integrand in Eq. (14) is small at frequencies $|f| \ll 1/t$ and the integral is dominated by the region around $f_* = 2/(\pi t)$ (with extent $\sim f_*$). f_* is of the order of $10 - 100\,\mathrm{kHz}$ for Hahn-echo sequences with total length $t = 10 - 100\,\mu\mathrm{s}$. If in this range of frequencies the noise spectra are of the form $S_{\mathrm{G}i}(f) = S_{\mathrm{G}i}^{\mathrm{hf}}(f_0/f)^\alpha$ $(0 < \alpha \leq 2$ typically), then:

$$\exp\left(-\frac{1}{2}\langle\delta\phi_E(t)^2\rangle\right) = \exp\left(-C_\alpha (2\pi t)^{\alpha+1} f_0^\alpha \sum_i D_{\mathrm{G}i}^2\, S_{\mathrm{G}i}^{\mathrm{hf}}\right) \equiv \exp\left[-\left(\frac{t}{T_2^{\mathrm{E}}}\right)^{\alpha+1}\right] , \tag{20}$$

where $C_\alpha = 2\sin(\frac{\alpha\pi}{2})(2^{1-\alpha} - 1)\Gamma(-1-\alpha)$, with Γ the Gamma function [23], and:

$$\frac{1}{T_2^{\mathrm{E}}} = 2\pi \left(C_\alpha f_0^\alpha \sum_i D_{\mathrm{G}i}^2\, S_{\mathrm{G}i}^{\mathrm{hf}}\right)^{\frac{1}{\alpha+1}} . \tag{21}$$

In the particular case $\alpha = 0.5$ (see main text), $C_{0.5} = \frac{4\sqrt{2\pi}}{3}(2^{1/2} - 1) \approx 1.38$, so that:

$$\frac{1}{T_2^{\mathrm{E}}} \approx 7.8 \left(f_0^{1/2} \sum_i D_{\mathrm{G}i}^2 \, S_{\mathrm{G}i}^{\mathrm{hf}} \right)^{2/3} . \tag{22}$$

The Hahn echo T_2^{E} and Ramsey $T_{2,\mathrm{hf}}^*$ [Eq. (18)] are thus proportional. Therefore, one would expect $T_{2,\mathrm{hf}}^* \simeq 50\,\mu$s at $\theta_{zx} = 99°$ where $T_2^{\mathrm{E}} \simeq 90\,\mu$s if the limiting noise mechanisms were the same at low and high frequency. The much shorter T_2^* measured in the present device hence support the existence of additional noise sources at low frequency.

C. CPMG sequence

For the more general CPMG sequence [21, 24] with noise spectra $S_{\mathrm{G}i}^{\mathrm{hf}}(f_0/f)^\alpha$ over extent $\sim 1/t$ around the frequency $f_{N_\pi} = N_\pi/(2t) \sim N_\pi/(2T_2^{\mathrm{CPMG}})$, we get the scaling

$$\langle \delta\phi^2(t) \rangle \sim t^{\alpha+1} N_\pi^{-\alpha} f_0^\alpha \sum_i D_{\mathrm{G}i}^2 \, S_{\mathrm{G}i}^{\mathrm{hf}} , \tag{23}$$

so that $\langle \delta\phi^2(t) \rangle \sim (t/T_2^{\mathrm{CPMG}})^{\alpha+1}$, with:

$$T_2^{\mathrm{CPMG}} \sim N_\pi^\gamma f_0^{-\gamma} \left(\sum_i D_{\mathrm{G}i}^2 \, S_{\mathrm{G}i}^{\mathrm{hf}} \right)^{-\frac{1}{\alpha+1}} \tag{24}$$

and $\gamma = \alpha/(\alpha+1)$, in agreement with Ref. 24.

S4. T_2^* IN THE NON ERGODIC REGIME

In order to measure $T_2^*(\theta_{zx})$, we record $\approx 5.5\,$s long Ramsey oscillations over one hour for each magnetic field orientation. We vary the acquisition time by averaging N consecutive traces ($t_{\mathrm{meas}} = N \times 5.5\,$s) and fit each of these data sets with a Gaussian decay where T_2^* is a free parameter. Since the acquisition time can be faster than the low-frequency noise correlation time τ (non ergodic regime), T_2^* is a stochastic variable that can be described by a statistical distribution [25].

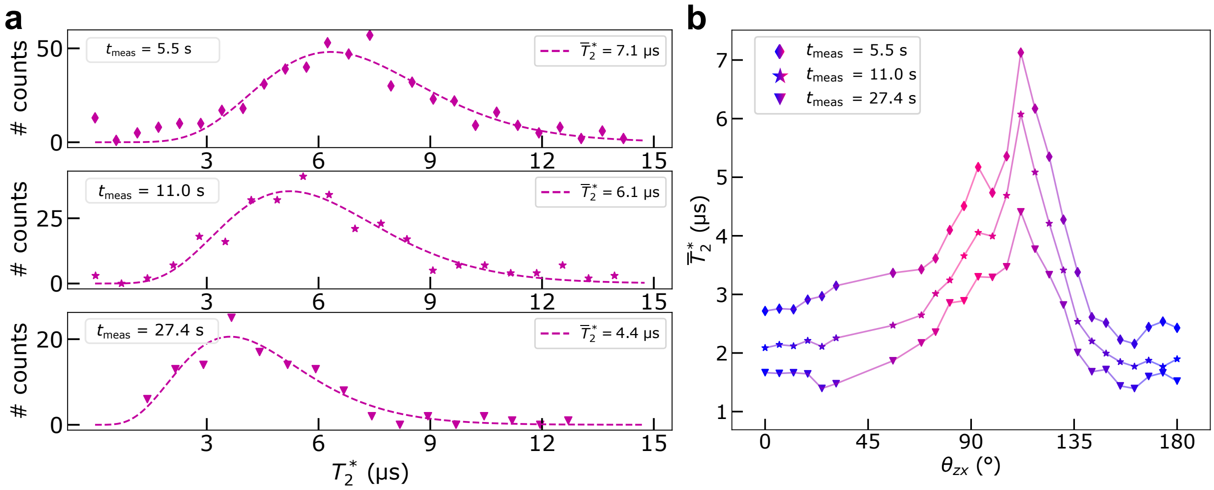

Figure S.4. **Spin coherence with correlated low-frequency noise (a)** T_2^* histograms for $t_{\mathrm{meas}} = 5.5\,$s ($N = 1$, diamonds), 11 s ($N = 2$, circles) and 27.4 s ($N = 5$, squares) at $\theta_{zx} = 111°$. The histograms are fitted with a Gamma distribution (dashed lines). **(b)** $\overline{T}_2^*$ extracted from the fits in (a) as a function of the magnetic field orientation θ_{zx}.

For $N \leq 5$, we can fit the histograms of T_2^* with a Gamma distribution as in Ref. [25] (see Fig S.4a):

$$f(T_2^*; \overline{T}_2^*, k) = \frac{k^k}{\overline{T}_2^{*k}\,\Gamma(k)} T_2^{*\,k-1} e^{-kT_2^*/\overline{T}_2^*} \tag{25}$$

where Γ is the Euler gamma function, $\overline{T}_2^*$ is the mean and k describes the shape (skewness) of the distribution. The thus extracted $\overline{T}_2^*$ is more robust to the presence of T_2^* data points far away from the mean, which are more frequent for small N's. In Fig S.4b, we plot the fitted $\overline{T}_2^*$ as a function of the magnetic field orientation for $N = 1$, 2, 5. The data exhibit a clear peak at $\theta_{zx} = 111°$, close to (but not exactly at) the sweet spot of $T_2^{\rm E}$.

For $N > 5$, the data set does not contain enough samples (less than 100) to extract the probability distribution parameters with high enough accuracy. In that case, we simply estimate $\overline{T}_2^*$ as the sample average of T_2^*. We point out that the distribution of T_2^*'s shall narrow when approaching the ergodic regime ($t_{\rm meas} \gg \tau$).

S5. HYPERFINE INTERACTION LIMIT FOR THE INHOMOGENEOUS DEPHASING TIME

The hyperfine interactions between the hole and the N nuclei spins are described by the following Hamiltonian [26, 27]:

$$H_{\rm int} = \frac{A}{2n_0} \sum_{n=1}^{N} \delta(\mathbf{r} - \mathbf{R}_n) \otimes \mathbf{J} \cdot \mathbf{I}_n \,, \tag{26}$$

where A is the hyperfine coupling constant, n_0 is the density of nuclei in the crystal, $\mathbf{I}_n$ is the spin operator of nuclei n at position $\mathbf{R}_n$, and $\mathbf{J}$ is the angular momentum operator acting on the $J = 3/2$ Bloch functions of the heavy and light holes (whereas the $\delta(\mathbf{r} - \mathbf{R}_n)$ acts on the envelopes). We discard here the small contributions from the split-off $J = 1/2$ components as well as the small $\propto J_x^3$, J_y^3, J_z^3 corrections arising from the cubic symmetry of the crystal [26].

Let $|\Uparrow\rangle$ and $|\Downarrow\rangle$ be the pseudo-spin states of the dot at a given magnetic field, and $|\psi_{\rm nucl}\rangle$ be the nuclear configuration. The first-order correction to the Larmor energy $\varepsilon_L = hf_L$ is:

$$\delta\varepsilon_L = \frac{A}{2n_0} \sum_{n=1}^{N} \langle\psi_{\rm nucl}|\, \mathbf{I}_n \,|\psi_{\rm nucl}\rangle \cdot \big(\langle\Uparrow|\, \delta(\mathbf{r} - \mathbf{R}_n) \otimes \mathbf{J} \,|\Uparrow\rangle - \langle\Downarrow|\, \delta(\mathbf{r} - \mathbf{R}_n) \otimes \mathbf{J} \,|\Downarrow\rangle \big) \,. \tag{27}$$

We next average over the nuclei configurations assuming uncorrelated and unpolarized nuclear spins with Gaussian-distributed quasi-static fluctuations [28]. The variance of $\delta\varepsilon_L$ is then:

$$\left\langle \delta\varepsilon_L^2 \right\rangle = \frac{A^2}{4n_0^2} \sum_{n=1}^{N} \left\langle I_x^2 \right\rangle \delta J_x^2(\mathbf{R}_n) + \left\langle I_y^2 \right\rangle \delta J_y^2(\mathbf{R}_n) + \left\langle I_z^2 \right\rangle \delta J_z^2(\mathbf{R}_n) \,, \tag{28}$$

where, for $\alpha \in \{x, y, z\}$:

$$\delta J_\alpha(\mathbf{R}_n) = \langle\Uparrow|\, \delta(\mathbf{r} - \mathbf{R}_n) \otimes J_\alpha \,|\Uparrow\rangle - \langle\Downarrow|\, \delta(\mathbf{r} - \mathbf{R}_n) \otimes J_\alpha \,|\Downarrow\rangle \;, \tag{29}$$

and $\left\langle I_x^2 \right\rangle = \left\langle I_y^2 \right\rangle = \left\langle I_z^2 \right\rangle = I(I+1)/3$. Taking a second average over nuclei spin distributions, and assuming slowly varying envelope functions, we reach:

$$\left\langle\left\langle \delta\varepsilon_L^2 \right\rangle\right\rangle = \frac{A^2}{12n_0} I(I+1)\nu \left(\overline{\delta J_x^2} + \overline{\delta J_y^2} + \overline{\delta J_z^2} \right) \,, \tag{30}$$

where ν is the fraction of nuclei carrying a spin, and:

$$\overline{\delta J_\alpha^2} = \int d^3\mathbf{R}\, \delta J_\alpha^2\,(\mathbf{R}) \;. \tag{31}$$

Finally, the rate of inhomogeneous dephasing due to hyperfine interactions is [29, 30]:

$$\Gamma_2^* = \frac{1}{T_2^*} = \frac{\sqrt{\langle\langle \delta\varepsilon_L^2 \rangle\rangle}}{\sqrt{2}\hbar} = \frac{|A|}{2\hbar} \sqrt{\frac{\nu I(I+1)}{6n_0}} \left(\overline{\delta J_x^2} + \overline{\delta J_y^2} + \overline{\delta J_z^2} \right)^{1/2} \;. \tag{32}$$

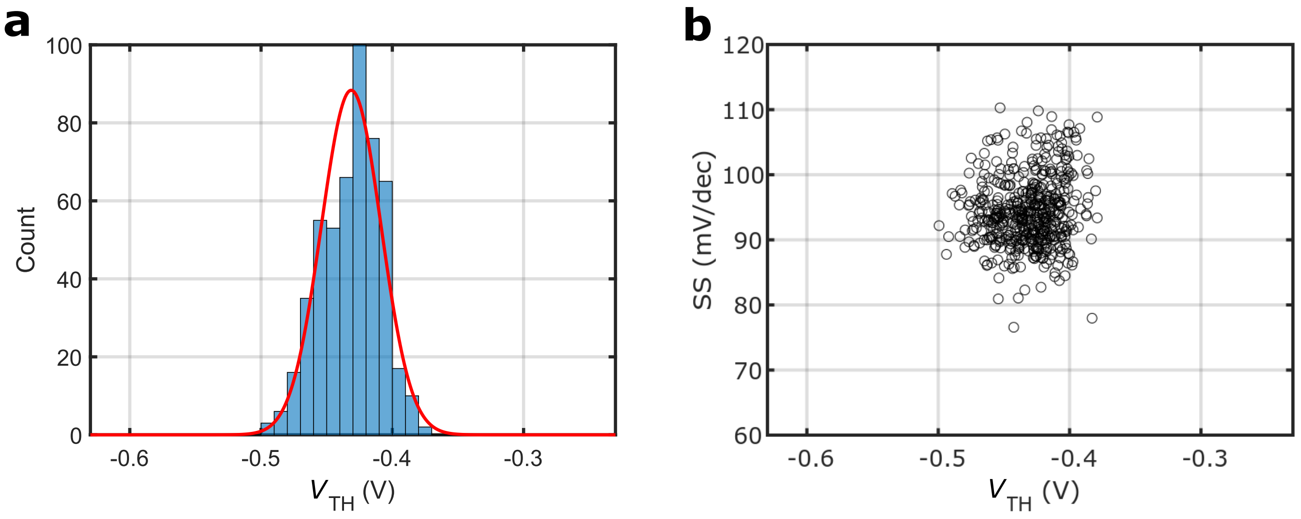

Figure S.5. **Yield across the full wafer. (a)** Distribution of the room temperature threshold voltages V_{TH} of all gates of the devices similar to the one measured in this paper (four 40 nm long gates separated by 40 nm, on top of a 17 nm thick and 100 nm wide channel, with a 6 nm thick SiO_2 gate oxide). All 3 other gates are biased at -2 V. The red curve is a gaussian fit with average 0.43 V and standard deviation 22 mV. V_{TH} is defined as the gate voltage where the derivative of the transconductance $\partial g_m/\partial V_{\mathrm{G}}$ is maximum. **(b)** Distribution of sub-threshold slope SS versus V_{TH} for all gates of all functional devices.

The above expression can be evaluated with the 6 bands $\mathbf{k}\cdot\mathbf{p}$ wave functions computed in section S1. For silicon, we use $n_0 = 49.94$ nm^{-3}, as well as $\nu = 4.7\%$, $I = 1/2$, and $|A| = 1.67\,\mu$eV for ^{29}Si isotopes [27]. This value of $|A|$ was specifically computed for holes with *ab initio* density functional theory [31]. The resulting T_2^*, plotted as a dashed line in Fig. 4 of the main text, is minimal when the magnetic field $\mathbf{B}$ is along y, and maximal when it is in the xz plane, as expected for a carrier that shows the strongest heavy-hole character when $\mathbf{J}$ is quantized along y. T_2^* is weakly dependent on the angle θ_{zx}, and is around $2.4\,\mu$s in the xz plane.

S6. UNIFORMITY AND QUALITY OF THE SAMPLES AT THE WAFER SCALE

The devices were extensively characterized at room temperature prior to low-temperature measurements. 90% of the 4-gate devices with 80 nm gate pitch (i.e. around 125 devices) are functional across the full 300 mm wafer. The devices are defined as functional according to 3 criteria:

- With any gate Gi closed ($V_{\mathrm{G}i} = +0.2$ V) and the other gates Gj open ($V_{\mathrm{G}j} = -2$ V, $j \neq i$), the source-drain current I_{D} must be lower than 10^{-11} A at source-drain bias $V_{\mathrm{DS}} = 50$ mV.
- With all gates open ($V_{\mathrm{G}i} = -2$ V), I_{D} must be greater than 10^{-7} A at $V_{\mathrm{DS}} = 50$ mV.
- The gate leakage current $I_{\mathrm{G}i}$ must be lower than 10^{-11} A.

Figure S.5a collects the room temperature threshold voltages V_{TH} measured for each gate of each functional device (with -2 V applied on the 3 other gates). Figure S.5b displays the sub-threshold slope (SS) versus the threshold voltage V_{TH} of each gate. The distribution of threshold voltages is sharply peaked around $V_{\mathrm{TH}} = -0.43$ V (standard deviation: 22 mV), which testifies the high uniformity of the devices at the wafer scale. As a comparison, the recent Ref. [32] reports a standard deviation of up to 145 mV for the first gate layer. The uniformity of the devices in the wafer is further supported by the narrow distribution of sub-threshold slopes.

As compared to Ref. [33], the fabrication process has been improved in several major aspects, that are described for instance in Ref. [34] (except for the exchange gates, that are not included in the present wafer):

- The source and drain are now doped *in situ* (during the overgrowth of the contacts). They were previously doped by ion implantation, which resulted in the spurious implantation of dopants in the channel.
- The source/drain junctions have been engineered to optimize the coupling with the reservoirs, including changes in the spacer design and thermal annealing step.

- High-k dielectrics (e.g. $HfSiO_2$) have been removed from the gate stack, leaving SiO_2 as the only gate oxide. High-k dielectrics are known to host higher densities of charge traps, which can be very detrimental in the few-hole regime [6].

- The silicon channel is thicker (17 nm) than in Ref. [33] (10 nm), which reduces the sensitivity to surface roughness [6].

---

[1] B. Venitucci, L. Bourdet, D. Pouzada, and Y.-M. Niquet, Electrical manipulation of semiconductor spin qubits within the g-matrix formalism, Physical Review B **98**, 155319 (2018).

[2] A. Crippa, R. Maurand, L. Bourdet, D. Kotekar-Patil, A. Amisse, X. Jehl, M. Sanquer, R. Laviéville, H. Bohuslavskyi, L. Hutin, S. Barraud, M. Vinet, Y.-M. Niquet, and S. D. Franceschi, Electrical spin driving by g-matrix modulation in spin-orbit qubits, Physical Review Letters **120**, 137702 (2018).

[3] V. P. Michal, B. Venitucci, and Y.-M. Niquet, Longitudinal and transverse electric field manipulation of hole spin-orbit qubits in one-dimensional channels, Physical Review B **103**, 045305 (2021).

[4] B. Voisin, V.-H. Nguyen, J. Renard, X. Jehl, S. Barraud, F. Triozon, M. Vinet, I. Duchemin, Y.-M. Niquet, S. de Franceschi, and M. Sanquer, Few-electron edge-state quantum dots in a silicon nanowire field-effect transistor, Nano Letters **14**, 2094 (2014).

[5] B. Venitucci and Y.-M. Niquet, Simple model for electrical hole spin manipulation in semiconductor quantum dots: Impact of dot material and orientation, Physical Review B **99**, 115317 (2019).

[6] B. Martinez and Y.-M. Niquet, Variability of electron and hole spin qubits due to interface roughness and charge traps, Physical Review Applied **17**, 024022 (2022).

[7] S.-D. Tzeng and S. Gwo, Charge trapping properties at silicon nitride/silicon oxide interface studied by variable-temperature electrostatic force microscopy, Journal of Applied Physics **100**, 023711 (2006).

[8] C. Kloeffel, M. J. Rančić, and D. Loss, Direct Rashba spin-orbit interaction in Si and Ge nanowires with different growth directions, Physical Review B **97**, 235422 (2018).

[9] S. D. Liles, F. Martins, D. S. Miserev, A. A. Kiselev, I. D. Thorvaldson, M. J. Rendell, I. K. Jin, F. E. Hudson, M. Veldhorst, K. M. Itoh, O. P. Sushkov, T. D. Ladd, A. S. Dzurak, and A. R. Hamilton, Electrical control of the g tensor of the first hole in a silicon MOS quantum dot, Physical Review B **104**, 235303 (2021).

[10] J. J. Pla, A. Bienfait, G. Pica, J. Mansir, F. A. Mohiyaddin, Z. Zeng, Y.-M. Niquet, A. Morello, T. Schenkel, J. J. L. Morton, and P. Bertet, Strain-induced spin-resonance shifts in silicon devices, Physical Review Applied **9**, 044014 (2018).

[11] B. Roche, E. Dupont-Ferrier, B. Voisin, M. Cobian, X. Jehl, R. Wacquez, M. Vinet, Y.-M. Niquet, and M. Sanquer, Detection of a large valley-orbit splitting in silicon with two-donor spectroscopy, Physical Review Letters **108**, 206812 (2012).

[12] V. P. Michal, J. C. Abadillo-Uriel, S. Zihlmann, R. Maurand, Y. M. Niquet, and M. Filippone, Tunable hole spin-photon interaction based on g-matrix modulation (2022), arXiv:2204.00404 [cond-mat.mes-hall].

[13] S. Bosco, B. Hetényi, and D. Loss, Hole spin qubits in Si FinFETs with fully tunable spin-orbit coupling and sweet spots for charge noise, PRX Quantum **2**, 010348 (2021).

[14] N. Ares, V. N. Golovach, G. Katsaros, M. Stoffel, F. Fournel, L. I. Glazman, O. G. Schmidt, and S. De Franceschi, Nature of tunable hole g factors in quantum dots, Physical Review Letters **110**, 046602 (2013).

[15] A. Abragam and B. Bleaney, *Electron paramagnetic resonance of transition ions* (Clarendon Press, Oxford, 1970).

[16] G. Ithier, E. Collin, P. Joyez, P. J. Meeson, D. Vion, D. Esteve, F. Chiarello, A. Shnirman, Y. Makhlin, J. Schriefl, and G. Schön, Decoherence in a superconducting quantum bit circuit, Physical Review B **72**, 134519 (2005).

[17] E. Paladino, Y. Galperin, G. Falci, and B. Altshuler, $1/f$ noise: Implications for solid-state quantum information, Reviews of Modern Physics **86**, 361 (2014).

[18] H. Y. Carr and E. M. Purcell, Effects of diffusion on free precession in nuclear magnetic resonance experiments, Physical Review **94**, 630 (1954).

[19] S. Meiboom and D. Gill, Modified spin-echo method for measuring nuclear relaxation times, Review of Scientific Instruments **29**, 688 (1958).

[20] L. M. K. Vandersypen and I. L. Chuang, NMR techniques for quantum control and computation, Reviews of Modern Physics **76**, 1037 (2005).

[21] L. Cywiński, R. M. Lutchyn, C. P. Nave, and S. Das Sarma, How to enhance dephasing time in superconducting qubits, Physical Review B **77**, 174509 (2008).

[22] M. J. Biercuk, A. C. Doherty, and H. Uys, Dynamical decoupling sequence construction as a filter-design problem, Journal of Physics B: Atomic, Molecular and Optical Physics **44**, 154002 (2011).

[23] F. W. Olver, D. W. Lozier, R. F. Boisvert, and C. W. Clark, *NIST Handbook of Mathematical Functions*, 1st ed. (Cambridge University Press, USA, 2010).

[24] J. Medford, L. Cywiński, C. Barthel, C. M. Marcus, M. P. Hanson, and A. C. Gossard, Scaling of dynamical decoupling for spin qubits, Physical Review Letters **108**, 086802 (2012).

[25] M. R. Delbecq, T. Nakajima, P. Stano, T. Otsuka, S. Amaha, J. Yoneda, K. Takeda, G. Allison, A. Ludwig, A. D. Wieck,

and S. Tarucha, Quantum dephasing in a gated GaAs triple quantum dot due to nonergodic noise, Physical Review Letters **116**, 046802 (2016).

[26] P. Machnikowski, K. Gawarecki, and L. Cywiński, Hyperfine interaction for holes in quantum dots: $k \cdot p$ model, Physical Review B **100**, 085305 (2019).

[27] S. Bosco and D. Loss, Fully tunable hyperfine interactions of hole spin qubits in Si and Ge quantum dots, Physical Review Letters **127**, 190501 (2021).

[28] I. A. Merkulov, A. L. Efros, and M. Rosen, Electron spin relaxation by nuclei in semiconductor quantum dots, Physical Review B **65**, 205309 (2002).

[29] J. Fischer, W. A. Coish, D. V. Bulaev, and D. Loss, Spin decoherence of a heavy hole coupled to nuclear spins in a quantum dot, Physical Review B **78**, 155329 (2008).

[30] C. Testelin, F. Bernardot, B. Eble, and M. Chamarro, Hole–spin dephasing time associated with hyperfine interaction in quantum dots, Physical Review B **79**, 195440 (2009).

[31] P. Philippopoulos, S. Chesi, and W. A. Coish, First-principles hyperfine tensors for electrons and holes in GaAs and silicon, Physical Review B **101**, 115302 (2020).

[32] A. M. J. Zwerver, T. Krähenmann, T. F. Watson, L. Lampert, H. C. George, R. Pillarisetty, S. A. Bojarski, P. Amin, S. V. Amitonov, J. M. Boter, R. Caudillo, D. Corras-Serrano, J. P. Dehollain, G. Droulers, E. M. Henry, R. Kotlyar, M. Lodari, F. Lüthi, D. J. Michalak, B. K. Mueller, S. Neyens, J. Roberts, N. Samkharadze, G. Zheng, O. K. Zietz, G. Scappucci, M. Veldhorst, L. M. K. Vandersypen, and J. S. Clarke, Qubits made by advanced semiconductor manufacturing, Nature Electronics **5**, 184 (2022).

[33] R. Maurand, X. Jehl, D. Kotekar-Patil, A. Corna, H. Bohuslavskyi, R. Laviéville, L. Hutin, S. Barraud, M. Vinet, M. Sanquer, and S. De Franceschi, A CMOS silicon spin qubit, Nature Communications **7**, 13575 (2016).

[34] T. Bédécarrats, B. C. Paz, B. M. Diaz, H. Niebojewski, B. Bertrand, N. Rambal, C. Comboroure, A. Sarrazin, F. Boulard, E. Guyez, J.-M. Hartmann, Y. Morand, A. Magalhaes-Lucas, E. Nowak, E. Catapano, M. Cassé, M. Urdampilleta, Y.-M. Niquet, F. Gaillard, S. De Franceschi, T. Meunier, and M. Vinet, A new FDSOI spin qubit platform with 40 nm effective control pitch, IEDM 10.1109/IEDM19574.2021.9720497 (2021).
